# Downregulation of SLC44A4 in nasopharyngeal carcinoma is associated with malignant progression, B-cell/TLS-related immune features, and sensitivity to DNA-damaging agents

**DOI:** 10.1371/journal.pone.0352812

**Published:** 2026-06-26

**Authors:** Qian Liu, Yiyue Yin, Guangxian Hu, Hongde Li, Min Li, Xiangjian Luo, Wenbin Liu

**Affiliations:** 1 Hunan Key Laboratory of Oncotarget Gene, Hunan Cancer Hospital and the Affiliated Cancer Hospital of Xiangya School of Medicine, Central South University, Changsha, Hunan, People’s Republic of China; 2 Key Laboratory of Carcinogenesis and Invasion, Chinese Ministry of Education, Cancer Research Institute, Xiangya School of Basic Medicine, Central South University, Changsha, Hunan, People’s Republic of China; 3 Department of Oncology, Nanjing Hospital of Chinese Medicine Affiliated to Nanjing University of Chinese Medicine, Nanjing, Jiangsu, People’s Republic of China; 4 The Hunan Provincial College of Clinical Laboratory, Changsha Medical University, Changsha, Hunan, People’s Republic of China; 5 Hunan Provincial Key Laboratory of the Traditional Chinese Medicine Agricultural Biogenomics, Changsha Medical University, Changsha, Hunan, People’s Republic of China; 6 Department of Pathology, Hunan Cancer Hospital and The Affiliated Cancer Hospital of Xiangya School of Medicine, Central South University, Changsha, Hunan, People’s Republic of China; Longgang Otorhinolaryngology Hospital & Shenzhen Key Laboratory of Otorhinolaryngology, Shenzhen Institute of Otorhinolaryngology, CHINA

## Abstract

Nasopharyngeal carcinoma (NPC) is characterized by aggressive progression, frequent metastasis, and heterogeneous therapeutic responses. Through integrated bulk transcriptomic datasets, single-cell analysis, clinical specimens, and NPC cell lines, we identified SLC44A4, a member of the choline transporter-like family, as a significantly downregulated gene in NPC. Transcriptomic analyses revealed that SLC44A4 expression was negatively associated with oxidative phosphorylation–related genes, DNA damage repair pathways, and malignant transcriptional programs related to proliferation, invasion, and metastasis. Moreover, SLC44A4-high tumors exhibited increased B-cell infiltration and enrichment of TLS-related transcriptional signatures. In vitro, SLC44A4 overexpression in NPC cells suppressed proliferation, colony formation, migration, and invasion, induced G0/G1 cell-cycle arrest, reduced expression of oxidative phosphorylation–related proteins, and selectively upregulated CXCL10. SLC44A4 overexpression also increased sensitivity to DNA-damaging agents, including temozolomide, doxorubicin, cisplatin, olaparib, and etoposide, while decreasing sensitivity to 5-fluorouracil. Together, these findings identify SLC44A4 as a potential tumor-suppressive factor in NPC and suggest that SLC44A4 may serve as a biomarker for metabolic state, B-cell/TLS-associated immune features, and vulnerability to DNA damage–based therapies.

## Introduction

Nasopharyngeal carcinoma (NPC) is a subtype of head and neck squamous cell carcinoma (HNSCC) with a distinct geographic distribution in southern China, Southeast Asia, and North Africa [1]. Although radiotherapy provides effective locoregional control for most patients with non-metastatic NPC, NPC has a particularly high propensity for distant metastasis among HNSCC subtypes [2,3]. Moreover, more than half of patients are diagnosed at advanced stages, and therapeutic options for recurrent or metastatic NPC (RM-NPC) are limited, often yielding poor outcomes [1,4]. Platinum-based combination chemotherapy remains the standard first-line treatment for RM-NPC; however, acquired resistance presents a significant barrier to long-term efficacy [5,6]. Concurrent chemoradiotherapy, although improving locoregional control, frequently leads to substantial toxicity, thereby compromising patients’ quality of life [7,8]. Despite some progress, emerging immunotherapies have shown only modest benefits in treating NPC [9–11]. Therefore, identifying novel vulnerabilities and actionable biomarkers is crucial for improving prognosis and enhancing therapeutic responses.

SLC44A4, also known as choline transporter-like protein 4 (CTL4), is a member of the SLC44 family (SLC44A1–5; CTL1–5). While the family is implicated in choline transport, only SLC44A1 and SLC44A2 have been experimentally confirmed to possess choline transport activity [12–14]. Whether SLC44A4 directly mediates choline transport therefore remains unproven, and its functions in tumor biology are poorly characterized. Other members of the SLC44 family, such as SLC44A1 and SLC44A2, have been associated with mitochondrial membrane metabolism, apoptosis resistance, and tumor growth [15–17], indicating a broader involvement of this family in metabolic and stress-adaptation programs. Given the growing recognition of solute carriers as key determinants of cancer metabolism and therapeutic response, elucidating the function of SLC44A4 represents an important and timely knowledge gap.

In this study, we demonstrate that SLC44A4 is significantly downregulated in NPC and is associated with clinical outcomes and immune microenvironmental features. Functionally, SLC44A4 suppresses malignant phenotypes and modulates cellular responses to anticancer agents. Together, these findings identify SLC44A4 as a potential prognostic biomarker and support its functional relevance to malignant progression and sensitivity to DNA damage-based therapies in NPC.

## Methods

### Patient cohorts

Public transcriptomic datasets of NPC from the GEO database were obtained from the National Center for Biotechnology Information Gene Expression Omnibus (GEO) (GSE13597, GSE53819, GSE102349) [18–20]. The limma package in R software (version 4.3.1) was used to determine the differential expression of mRNAs. |log2FC| > 1 and FDR < 0.05 were defined as the thresholds for screening differential expression of mRNAs. Survival analyses of SLC44A4 in patients with HNSCC were performed using the GEPIA2 database (http://gepia2.cancer- pku.cn) [21]. Hazard ratios from Cox proportional hazards analysis and Kaplan-Meier curves were then obtained from this website.

Single-cell RNA (scRNA) data were obtained from the Genome Sequence Archive of the BIG Data Center at the Beijing Institute of Genomics, Chinese Academy of Sciences, under accession number HRA000087 (accessible at http://bigd.big.ac.cn/gsa-human) [22]. The samples used in this study included primary tumors or lymph node metastases from 7 EBV + NPC patients, and 7 non-cancerous nasopharyngeal samples from normal subjects. Downloaded FASTQ files were subsequently analyzed using the Seurat package (5.0.1) after obtaining downstream analysis files through the CellRanger (2.1.0) process. Cell screening criteria were as follows: nCount_RNA >= 500 & nFeature_RNA < 5000 & nFeature_RNA >= 700 & percent.mt < 0.20 & log10GenesPerUMI > 0.8, and 124,780 cells were finally retained.

### Gene set enrichment analysis

KEGG pathway enrichment analysis [23], Gene Set Enrichment Analysis (GSEA) and gene set variation analysis (GSVA) were performed with the R package “clusterProfiler” [24] based on gene sets of MsigDB. GSEA is an algorithm that can be applied for the analysis of functional differences by focusing on gene sets [25]. Functional gene expression signatures (Fges) were applied for an overview of the profile of immune characteristics [26]. In the present work, NPC samples were divided into two groups according to the expression level according to SLC44A4 expression, namely, the high-expression group and the low-expression group. The number of gene set permutations was set to 1000. The enriched pathways for each phenotype were ranked according to the nominal P value and the normalized enrichment score (NES).

### Weighted gene Co-expression network analysis

Gene co-expression networks were constructed using the WGCNA R package (version 1.73) [27]. Initial data quality control was performed using the “goodSamplesGenes” function to remove genes and samples with excessive missing values or zero variance. A signed co-expression network was constructed to distinguish positive and negative correlations. The soft-thresholding power (β) was determined through analysis of scale-free topology fit. Specifically, we tested powers from 1 to 20 and selected β = 6 based on achieving a scale-free topology fit index (R²) of approximately 0.9. Co-expression modules were identified using dynamic tree cutting with the following parameters: minimum module size = 30 genes, deepSplit = 2, and pamRespectsDendro = FALSE. Module eigengenes (MEs) were calculated as the first principal component of each module’s expression matrix. To reduce redundancy, highly correlated modules were merged using a cut height of 0.25 on the module eigengene dendrogram, corresponding to a correlation threshold of >0.75. This resulted in 46 distinct co-expression modules, each assigned a unique color label. Module functional significance was assessed through enrichment analysis against established gene signatures. Specifically, Tertiary lymphoid structures (TLS)-related modules were identified based on significant overlap with known TLS signature genes (CXCL13, MS4A1, CD79A, CD19, etc.).

### Cell lines

HONE1, SUNE1 cells were grown in RPMI-1640 (Gibco BRL) media supplemented with 10% v/v heat-inactivated fetal bovine serum (Invitrogen, Carlsbad, CA, USA). All cancer cell lines were obtained from the Cancer Research Institute of Central South University. All the cell lines involved were cultured at 37 °C in a humidified incubator containing 5% CO2.

### Statistical analysis

Statistical analyses were performed using the R software (V.4.3.1, R Core Team, Foundation for Statistical Computing, Vienna, Austria). Wilcoxon rank-sum test and chi-square test were conducted for continuous and categorical variables, respectively. For all analyses, a two-tailed P value of < 0.05 was considered statistically significant. *P < 0.05, **P < 0.01, ***P < 0.001, ****P < 0.0001.

## Results

### 1. Choline metabolism–related genes are associated with malignant progression in nasopharyngeal carcinoma

**Fig 1:**
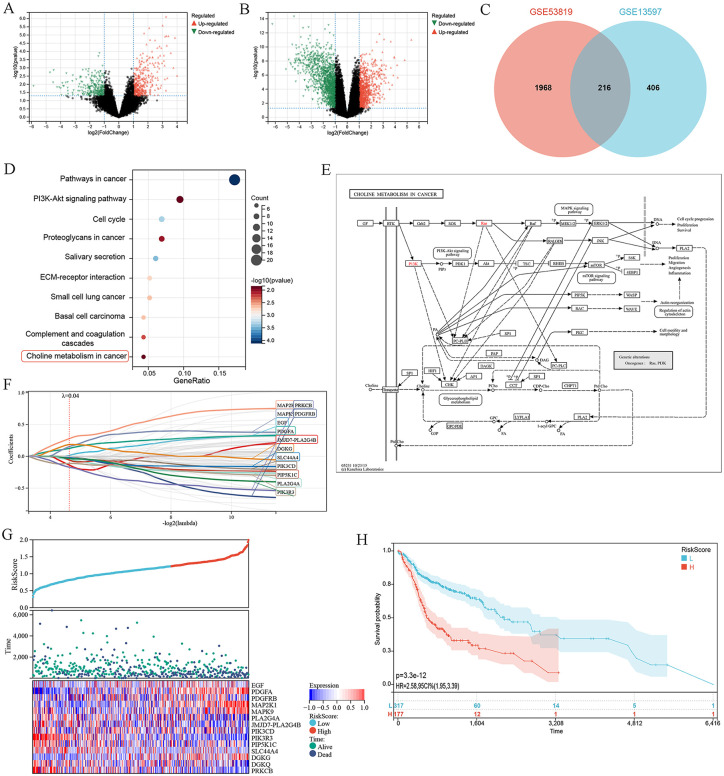
Identification of choline metabolism–related genes associated with malignant progression and prognosis. **(A–B)** Differentially expressed genes (DEGs) between tumor and non-tumor tissues in the GEO datasets GSE13597 (A) and GSE53819 **(B)**, identified using a threshold of |log₂ fold change| > 1; **(C)** Venn diagram showing 216 DEGs shared between the two NPC datasets; **(D)** KEGG pathway enrichment analysis of the shared DEGs; **(E)** Overview of genes involved in the KEGG choline metabolism pathway evaluated in the TCGA-HNSC cohort; **(F)** LASSO–Cox regression analysis identifying 14 choline metabolism–related genes used to construct a prognostic risk-score model; **(G)** Distribution of risk scores and patient stratification into high- and low-risk groups based on the optimal cutoff value; **(H)** Kaplan–Meier survival analysis showing significantly poorer overall survival in the high-risk group.

To delineate molecular alterations underlying nasopharyngeal carcinoma (NPC), we conducted differential expression analyses between tumor and adjacent non-tumor tissues using two independent GEO datasets (GSE13597 and GSE53819). Applying a threshold of |log₂ fold change| > 1, we identified 622 and 2,184 differentially expressed genes (DEGs), respectively (Fig 1A–B). Cross-dataset intersection analysis revealed 216 consistently dysregulated genes common to both cohorts (Fig 1C). Subsequent KEGG pathway enrichment analysis of these overlapping DEGs highlighted “Choline metabolism in cancer” as one of the top ten significantly enriched pathways (Fig 1D).

Given that NPC represents a distinct clinicopathological subtype of head and neck squamous cell carcinoma (HNSCC), we further assessed the prognostic relevance of genes within the KEGG choline metabolism pathway using the TCGA-HNSC cohort (Fig 1E). LASSO–Cox regression analysis identified 14 candidate genes, which were utilized to construct a prognostic risk-score model (Fig 1F). Based on an optimal risk-score cutoff (1.22), patients were stratified into high- and low-risk subgroups (Fig 1G). Kaplan–Meier survival analysis, supplemented by Cox proportional hazards regression, demonstrated significantly worse overall survival in the high-risk group (Fig 1H).

### 2. SLC44A4 is downregulated in NPC and correlates with patient prognosis

**Fig 2:**
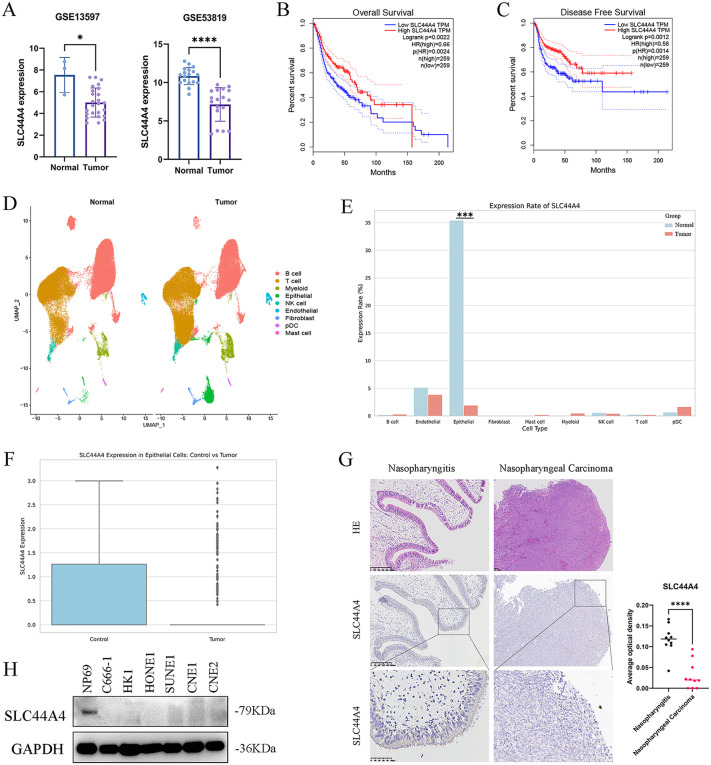
Downregulation of SLC44A4 in NPC and its association with patient prognosis. **(A)** Expression patterns of the 14 LASSO-selected genes in the two NPC cohorts (GSE13597 and GSE53819), identifying SLC44A4 as the only gene consistently downregulated across both datasets; **(B–C)** Kaplan–Meier analyses showing that higher SLC44A4 expression is associated with improved overall survival (B) and disease-free survival (C) in the TCGA-HNSC cohort; **(D)** Schematic overview of the single-cell RNA-sequencing dataset, including EBV-positive NPC primary tumors, lymph node metastases, and non-malignant nasopharyngeal tissues; **(E–F)** Single-cell expression analysis showing that SLC44A4 is predominantly expressed in non-malignant epithelial cells and markedly reduced in malignant epithelial cells; **(G)** Immunohistochemical analysis demonstrating lower SLC44A4 protein expression in NPC tissues compared with nasopharyngitis controls; **(H)** Western blot analysis showing high SLC44A4 expression in NP69 cells and near-undetectable expression across six NPC cell lines.

Among the 14 genes selected by LASSO, SLC44A4 was the only gene that showed consistent differential expression across both NPC datasets (GSE13597 and GSE53819) (Fig 2A). In the TCGA-HNSC cohort, elevated SLC44A4 expression was significantly associated with improved overall survival and disease-free survival (Fig 2B–C), underscoring its potential role as a favorable prognostic marker.

To characterize SLC44A4 expression within the tumor microenvironment (TME), we interrogated a scRNA-seq dataset derived from seven NPC patients and seven non-malignant nasopharyngeal tissue controls (Fig 2D). SLC44A4 expression was predominantly localized to non-malignant epithelial cells and was significantly reduced in malignant epithelial cells from NPC samples (Fig 2E–F). Consistent with this, immunohistochemical analysis of clinical specimens revealed markedly lower SLC44A4 protein levels in NPC tissues compared to nasopharyngitis controls (Fig 2G). Furthermore, Western blot analysis confirmed high SLC44A4 expression in the immortalized nasopharyngeal epithelial cell line NP69, whereas its expression was nearly undetectable across six established NPC cell lines (Fig 2H). These multi-level findings collectively demonstrate pervasive downregulation of SLC44A4 in NPC.

### 3. SLC44A4 is associated with mitochondrial energy metabolism and malignant phenotype in NPC

**Fig 3:**
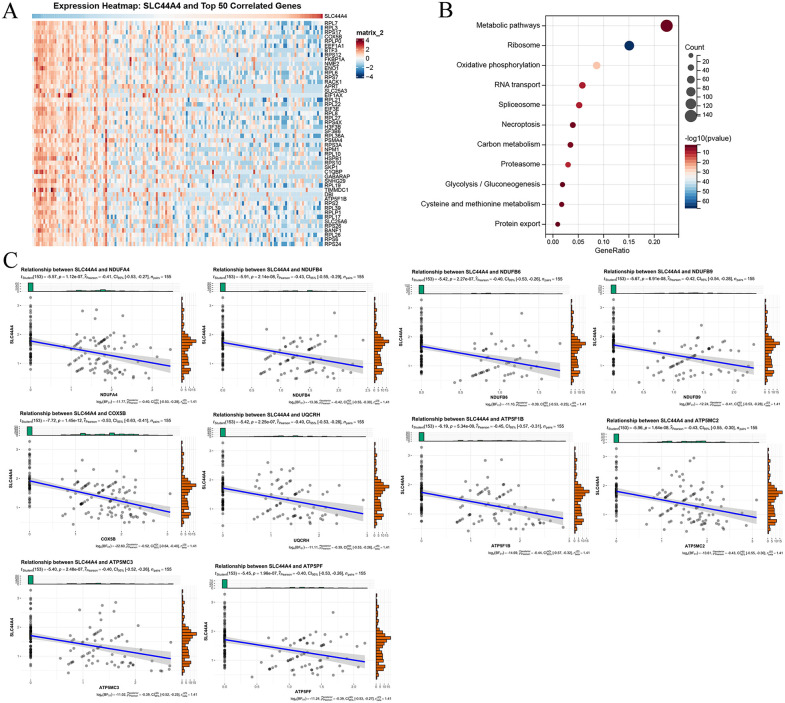
Correlation and pathway enrichment analysis of SLC44A4-associated genes at the sin-gle-cell level. **(A)** Heatmap showing the top 50 genes with the most significant correlation with SLC44A4 expression; **(B)** KEGG pathway enrichment analysis of these SLC44A4-correlated genes, revealing significant enrichment in pathways associated with central carbon metabolism and energy production; **(C)** SLC44A4 expression is negatively correlated with the mRNA levels of multiple core subunit genes of mitochondrial OXPHOS complexes, including complexes I, III, IV, and V.

To investigate the functional role of SLC44A4, we performed gene–gene correlation analysis in epithelial cells derived from the single-cell RNA-seq dataset. SLC44A4 expression exhibited a negative correlation with 1,186 genes and a positive correlation with 199 genes (Fig 3A). KEGG enrichment analysis of genes co-expressed with SLC44A4 revealed predominant involvement in metabolic pathways, with significant enrichment in central carbon and energy metabolism, including oxidative phosphorylation (OXPHOS), carbon metabolism, and glycolysis/gluconeogenesis (Fig 3B). Notably, among the top 100 genes most strongly correlated with SLC44A4, ten encode core subunits of the OXPHOS machinery and were all negatively correlated with SLC44A4 expression. These genes span multiple OXPHOS complexes, including complex I (NDUFB4, NDUFB9, NDUFA4, NDUFB6), complex III (UQCRH), complex IV (COX5B), and ATP synthase/complex V (ATP5F1B, ATP5MC2, ATP5MC3, ATP5PF) (Fig 3C).

**Fig 4:**
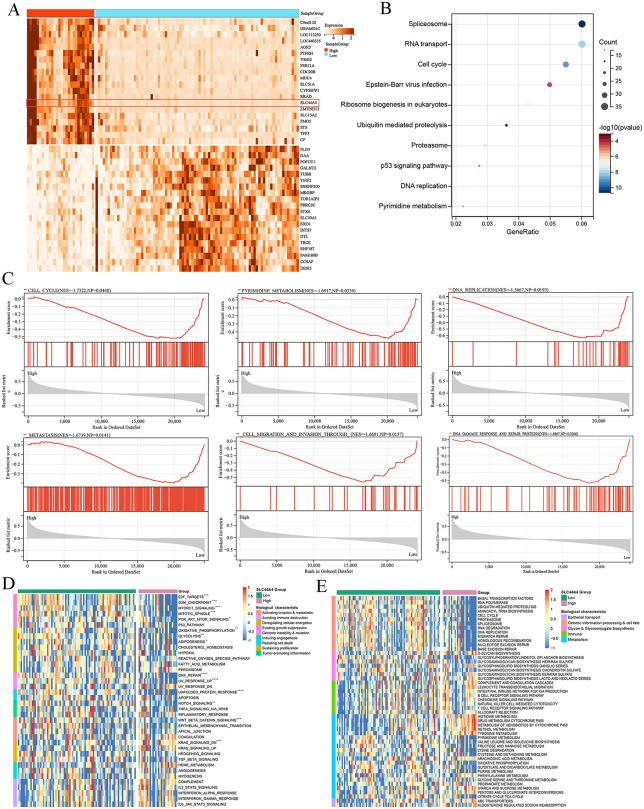
Functional implications of SLC44A4 expression in NPC. **(A)** Stratification of NPC patients in the GSE102349 cohort into SLC44A4-high and SLC44A4-low groups based on gene expression levels; **(B–C)** KEGG and GSEA showing enrichment of pathways related to cell cycle progression, proliferation, invasion, and metastasis in the SLC44A4-low group; **(D)** Hallmark pathway activity differences between the SLC44A4-low and SLC44A4-high groups; **(E)** KEGG-based biological characteristics between low- and high- SLC44A4 expression groups.

We next extended these observations to an independent bulk-transcriptomic NPC cohort. In the NPC cohort (GSE102349, n = 113), patients were stratified into SLC44A4-high and -low groups based on expression levels (Fig 4A). KEGG and GSEA revealed significant enrichment of pathways related to cell cycle progression, proliferation, invasion, and metastasis in the SLC44A4-low group, suggesting a suppressive role for SLC44A4 in malignant progression (Fig 4B-C). GSVA using Hallmark gene sets [28] further demonstrated that the SLC44A4-high group exhibited attenuated proliferative programs, along with reduced signatures of oxidative phosphorylation (OXPHOS) and glycolysis. Notably, signatures related to DNA damage repair and UV response were also diminished in the SLC44A4-high group (A – S1 Table; Fig 4D).

To obtain a more comprehensive functional view, we performed pathway-level analysis using the KEGG Legacy database (B – S1 Table; Fig 4E). The results showed that elevated SLC44A4 expression was linked to broad downregulation of genetic information processing systems, raising the possibility that SLC44A4-high tumors may be more vulnerable to DNA damage–based therapies. Concurrently, metabolic programs were substantially remodeled, characterized by activation of biotransformation and detoxification pathways, alongside suppression of core energy metabolism, amino acid catabolism, and nucleotide biosynthesis. Furthermore, high SLC44A4 expression correlated with an enhanced immune-activated state and with alterations in glycan and glycoconjugate biosynthesis (Fig 4E).

### 4. SLC44A4-high tumors are characterized by TLS/B-cell-enriched TME

**Fig 5:**
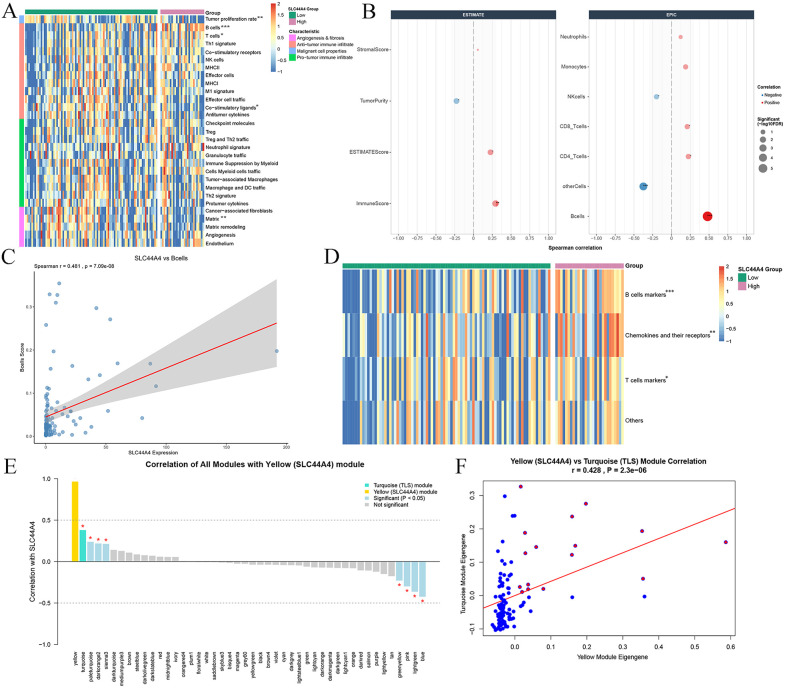
Association between SLC44A4 expression and an immune-activated, TLS/B-cell-enriched TME. **(A)** Fges analysis showing reduced tumor proliferation and stromal activation but enhanced immune cell functional activity—particularly B-cell activity—in SLC44A4-high tumors; **(B)** Correlations between SLC44A4 expression and ESTIMATE-derived ImmuneScore, ESTIMATEScore, and tumor purity; **(C)** EPIC deconvolution analysis demonstrating a strong positive correlation between SLC44A4 expression and B-cell infiltration; **(D)** GSVA based on established tertiary lymphoid structure (TLS) gene signatures showing increased TLS activity, immune cell activation, and chemokine/chemokine receptor signaling in the SLC44A4-high group; **(E–F)** WGCNA identifying a strong positive correlation between the SLC44A4-containing yellow module and the TLS-associated turquoise module, indicating coordinated transcriptional regulation. Fges, functional gene expression signatures; TME, Tumor microenvironment.

Given the observed association between SLC44A4 expression and immune activation, we next characterized the functional landscape of the TME. Using the functional gene expression signature (Fges) framework developed by Bagaev et al. [26], which enables systematic quantification of tumor, immune, and stromal activities, we found that SLC44A4-high tumors exhibited lower tumor proliferation and stromal activation scores, but displayed enhanced immune cell functional activity—particularly in B cells—suggesting that SLC44A4 expression correlates with an immune permissive TME (C – S1 Table; Fig 5A). Consistent with this, ESTIMATE analysis demonstrated that SLC44A4 expression was positively correlated with ImmuneScore and ESTIMATEScore, while negatively correlated with tumor purity (Fig.5B). EPIC deconvolution further supported these findings, revealing a strong positive correlation between SLC44A4 expression and B-cell infiltration (Fig 5B–C).

Tertiary lymphoid structures (TLS) constitute a pivotal tissue microenvironment that facilitates efficient anti-tumor immune responses mediated by B cells [29]. To explore the relationship between SLC44A4 expression and TLS-related immune features, we performed bioinformatic analyses using established TLS gene signatures (D – S1 Table) [30–32]. GSVA revealed that, compared with the SLC44A4-low group, the SLC44A4-high group exhibited significantly elevated TLS activity, accompanied by enhanced immune cell function and upregulated chemokine/chemokine receptor sig-naling (Fig 5D)—a pathway known to critically drive TLS formation [30].

We then applied WGCNA to examine coordinated transcriptional programs. WGCNA identified 46 co-expression modules (S1A–B Fig). SLC44A4 was assigned to the yellow module (S1C Fig), whereas TLS signature genes were concentrated in the turquoise module. Among all module–module interactions involving the yellow, SLC44A4-containing module, the turquoise, TLS-associated module showed the strongest positive correlation with it (r = 0.428, p = 2.3 × 10 ⁻ ⁶) (E – S1 Table; Fig 5E–F). These results indicate robust co-variation between SLC44A4-related transcriptional programs and TLS-associated activity. Together, these integrated analyses suggest that high SLC44A4 expression delineates a TLS-enriched microenvironment characterized by a B-cell-dominant functional state.

### 5. SLC44A4 overexpression suppresses malignant phenotypes and alters metabolic and chemokine-related programs in NPC cells

**Fig 6:**
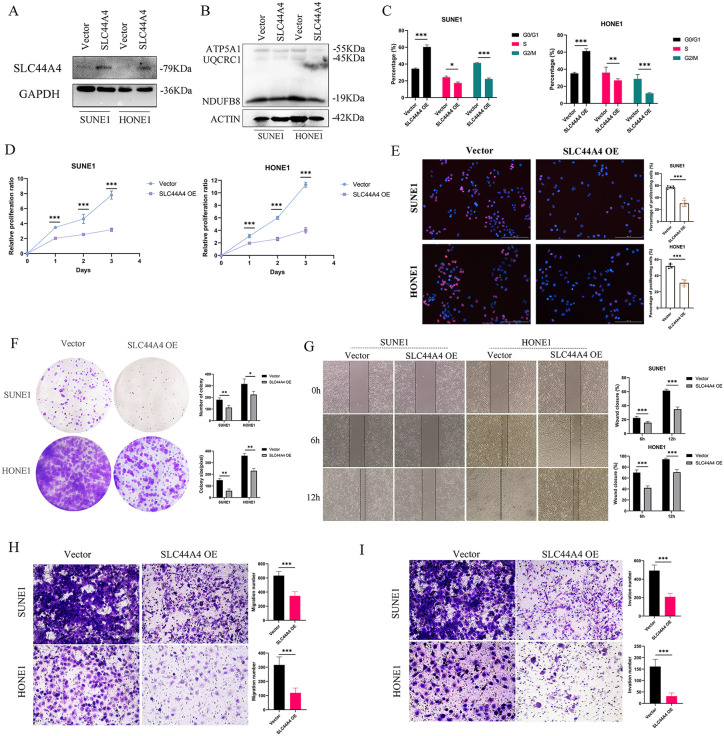
Functional effects of SLC44A4 overexpression on NPC cell lines. **(A)** Validation of SLC44A4 overexpression in SUNE1 and HONE1 cell lines; **(B)** SLC44A4 overexpression reduced the protein levels of representative oxidative phosphorylation subunits, including NDUFB8, UQCRC1, and ATP5A1, corresponding to mitochondrial complexes I, III, and V, respectively; **(C)** Flow cytometric analysis showing increased G0/G1-phase arrest and reduced G2/M-phase population upon SLC44A4 overexpression; **(D–E)** CCK-8 and EdU incorporation assays demonstrating reduced cell viability and proliferation in SLC44A4-overexpressing cells; **(F)** Colony formation assays showing fewer and smaller colonies following SLC44A4 overexpression; **(G)** Wound-healing assays indicating impaired migratory capacity in SLC44A4-overexpressing NPC cells; **(H–I)** Transwell migration (H) and invasion (I) assays confirming suppression of cell motility and invasiveness by SLC44A4.

To functionally validate the role of SLC44A4 in NPC, we established stable SLC44A4 overexpressing models in SUNE1 and HONE1 cell lines (Fig 6A). We first examined whether SLC44A4 overexpression affected the expression of selected genes involved in choline and phospholipid metabolism [33]. qPCR analysis showed that PLD1 and PLCL2 were the most consistently induced genes following SLC44A4 overexpression, whereas the remaining genes exhibited only modest or cell line-dependent changes (S2A–B Fig). Consistent with our bioinformatic correlation analyses, SLC44A4 overexpression also markedly decreased the protein levels of several oxidative phosphorylation-related subunits, including NDUFB8, UQCRC1, and ATP5A1, which represent mitochondrial complexes I, III, and V, respectively (Fig 6B).

We next investigated whether SLC44A4 directly affected the expression of chemokines implicated in TLS formation and immune cell recruitment, including CXCL13, CCL19, CCL21, CXCL12, CXCL9, CXCL10, and CXCL11 [34]. Notably, SLC44A4 overexpression selectively and significantly induced CXCL10 mRNA expression in NPC cells, whereas the other chemokines showed no consistent upregulation across the tested cell lines (S2C–D Fig).

At the phenotypic level, flow cytometry showed that SLC44A4 overexpression increased the proportion of cells in G0/G1 and decreased the G2/M fraction, indicating cell-cycle arrest (Fig 6C). CCK-8 and EdU assays further demonstrated reduced cell viability and proliferation upon SLC44A4 overexpression (Fig 6D–E). Consistently, colony formation assays revealed that SLC44A4 overexpressing cells formed fewer and smaller colonies compared with controls (Fig 6F). Wound-healing assay demonstrated that SLC44A4 overexpression significantly inhibited the migratory capacity of NPC cells (Fig 6G). Furthermore, Transwell assays revealed that SLC44A4 overexpression significantly impaired both migratory (Fig 6H) and invasive (Fig 6I) capabilities. Collectively, these results suggest that restoring SLC44A4 suppresses multiple malignant phenotypes in NPC cells.

### 6. SLC44A4 enhances cellular sensitivity to DNA damage-inducing therapeutic agents

Given that GSVA revealed a strong negative association between high SLC44A4 expression and pathways related to genomic stability and DNA damage response, we hypothesized that SLC44A4 expression may confer a specific therapeutic vulnerability to DNA damage–based treatments. To test this hypothesis, we selected a panel of antitumor agents with complementary mechanisms of action, including topoisomerase inhibitors doxorubicin (which is accompanied by reactive oxygen species [ROS] generation) and etoposide (with minimal ROS contribution); cisplatin, an inducer of DNA interstrand crosslinks; temozolomide, a DNA-alkylating agent; olaparib, a PARP inhibitor targeting DNA damage repair pathways; and 5-FU, an antimetabolite that interferes with DNA synthesis.

**Fig 7:**
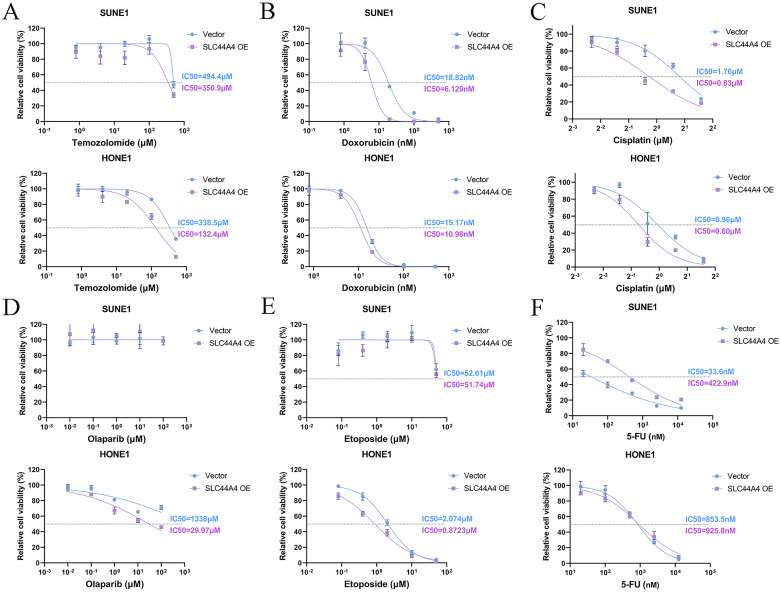
Effects of SLC44A4 on NPC cell sensitivity to DNA damage–based therapies. **(A–E)** Cell viability assays showing increased sensitivity of SLC44A4-overexpressing NPC cells to temozolomide **(A)**, doxorubicin **(B)**, cisplatin **(C)**, olaparib **(D)**, and etoposide **(E)**; **(F)** SLC44A4 overexpression reduces sensitivity to 5-fluorouracil (5-FU).

The results showed that SLC44A4 overexpression significantly increased the sensitivity of NPC cells to temozolomide (Fig 7A), doxorubicin (Fig 7B), cisplatin (Fig 7C), olaparib (Fig 7D), and etoposide (Fig 7E). In contrast, SLC44A4 overexpression reduced sensitivity to 5-FU, indicating increased tolerance to antimetabolite-based therapy (Fig 7F). These findings demonstrate that SLC44A4 enhances responsiveness to multiple DNA damage-inducing agents while reducing sensitivity to antimetabolite treatment, supporting its potential utility as a biomarker of therapeutic vulnerability in NPC.

## Discussion

In this study, we integrated bulk transcriptomic analyses, single-cell profiling, clinical outcome analyses, and functional assays to characterize the role of SLC44A4 in NPC. We demonstrate that SLC44A4 is markedly downregulated in NPC and that reduced expression is associated with unfavorable clinical outcomes. SLC44A4 overexpression suppressed multiple malignant phenotypes, including proliferation, migration, and invasion, and selectively enhanced cellular sensitivity to several DNA damage-inducing agents.

Although SLC44A4 is annotated as a choline transporter-like protein, its direct choline transport activity in NPC remains unproven [12–14]. In the present study, our qPCR analysis showed that SLC44A4 overexpression consistently upregulated PLD1 and PLCL2 in both HONE1 and SUNE1 cells, suggesting a potential link between SLC44A4 and choline/phospholipid metabolic regulation. However, these data do not directly establish SLC44A4 as a functional choline transporter. Future studies using radiolabeled or stable isotope-labeled choline uptake assays, choline flux analysis, and lipidomic profiling are required to determine whether SLC44A4 directly regulates choline transport or instead modulates choline/phospholipid metabolism indirectly.

Emerging evidence suggests that solute carrier (SLC) transporters influence therapeutic response not only by regulating drug uptake but also by modulating nutrient availability, redox balance, and stress-adaptation pathways [35]. In the present study, SLC44A4 overexpression did not induce broad chemosensitization. Instead, it exposed a selective vulnerability to genotoxic stress, as reflected by increased sensitivity to cisplatin, doxorubicin, etoposide, temozolomide, and the PARP inhibitor olaparib, while reducing sensitivity to the antimetabolite 5-fluorouracil. Notably, the differential responses to olaparib across cell models and following SLC44A4 overexpression suggest that this vulnerability may reflect altered DNA damage tolerance or repair capacity rather than dependence on a single DNA repair pathway. In contrast, the reduced responsiveness to 5-fluorouracil is consistent with a metabolically restrained or slower-cycling cellular state, arguing against nonspecific cytotoxic effects.

Mechanistically, this phenotype may be linked to suppression of mitochondrial oxidative phosphorylation-related programs. SLC44A4 overexpression markedly decreased the levels of several oxidative phosphorylation-related proteins, and mitochondrial respiration is intimately connected to redox homeostasis, reactive oxygen species generation, and the bioenergetic support required for DNA damage repair [36,37]. Thus, SLC44A4 may increase the susceptibility of NPC cells to DNA damage-based therapies by perturbing mitochondrial oxidative metabolism and redox balance. In addition, as a member of the choline transporter-like protein family, SLC44A4 may alter choline metabolism, phospholipid homeostasis, and membrane remodeling, processes that can shape cellular adaptation to chemotherapy-induced stress [38]. Further studies are needed to define how SLC44A4 mechanistically connects choline/phospholipid metabolism, mitochondrial function, and DNA damage responses in NPC.

Beyond tumor cell–intrinsic phenotypes, our analyses uncovered a strong association between high SLC44A4 expression and an immune-activated TME characterized by enhanced B-cell activity and enrichment of TLS-related signatures. Accumulating evidence suggests that tumor-infiltrating B cells and TLSs are associated with improved clinical outcomes and better therapy responses in various tumor types [29]. Recent work in NPC reported that recurrent disease exhibits decreased B-cell abundance and TLS formation and that these features may predict survival [4,39]. These findings suggest that SLC44A4 may be associated with antitumor immune remodeling in NPC. However, the association between SLC44A4 and TLS/B-cell-enriched immune features was mainly inferred from transcriptomic analyses. Therefore, spatial transcriptomics, multiplex immunohistochemistry or immunofluorescence, and histological annotation of TLS maturation states will be required to validate whether SLC44A4-high tumors are indeed enriched for spatially organized TLSs.

qPCR analysis showed that SLC44A4 overexpression significantly increased CXCL10 expression, whereas CXCL13, CCL19, CCL21, and CXCL12 did not display consistent upregulation. The selective induction of CXCL10 suggests that SLC44A4 may contribute to an inflammatory chemokine milieu through the CXCL10–CXCR3 axis. Although the CXCL10–CXCR3 axis has been implicated in TLS-associated inflammation and B cell-mediated antitumor immunity [40–42], the organization and maturation of TLSs largely depend on classical lymphoid organogenesis modules, including CXCL13–CXCR5, CCL19/CCL21–CCR7, LTβR–NF-κB signaling, HEVs, and FDCs [34]. Thus, CXCL10 is more likely to function as an amplifier of TLS-related immune activity rather than as an autonomous TLS-organizing factor. Consistent with this interpretation, SLC44A4 does not appear to directly activate a canonical TLS/B-cell chemokine program in NPC tumor cells. Instead, its association with B-cell/TLS-enriched immune features may reflect a more immune-permissive tumor state or indirect crosstalk with stromal and immune cells.

From a translational medicine perspective, SLC44A4 expression may have potential value in biomarker-guided therapeutic strategies. Our data raise the possibility that patients with high SLC44A4 expression may be more responsive to DNA damage–targeting treatments, such as platinum-based chemotherapy, radiotherapy, or DNA damage–based combination strategies but may derive less benefit from fluoropyrimidine-based treatment such as 5-FU. In addition, the association between SLC44A4 expression and B-cell/TLS-related immune features suggests that this gene may be relevant to immune microenvironment-based stratification. However, these possibilities require further validation. Prospective clinical cohorts incorporating treatment response data, survival outcomes, standardized methods for SLC44A4 detection, and predefined cutoff values will be necessary before SLC44A4 can be applied to guide individualized treatment in clinical practice.

Notably, pharmacological induction of SLC transporters has been demonstrated in clinically relevant contexts [43]. For instance, epigenetic modulation using the HDAC inhibitor vorinostat was explored to increase SLC6A2/NET expression to enhance therapeutic targeting, supporting the feasibility of transcriptional reactivation of SLC transporters [44]. These observations highlight the importance of elucidating the epigenetic and transcriptional mechanisms underlying SLC44A4 downregulation in NPC. A deeper understanding of these regulatory layers may inform future strategies aimed at restoring SLC44A4 expression and exploiting its tumor-suppressive and immunomodulatory potential.

In summary, the convergence of multi-layered molecular, functional, and clinical observations supports SLC44A4 as a potential tumor-suppressive factor associated with malignant phenotypes in NPC. Moreover, SLC44A4 may serve as a predictive biomarker for sensitivity to DNA damage–based therapies. With a deeper understanding of its biological functions and regulatory mechanisms, SLC44A4-based biomarker stratification may help refine future therapeutic strategies for NPC.

## Supporting information

S1 Table TLS functional genes and GSVA scores of NPC patients based on Hallmark, KEGG, Fges, and TLS gene sets. (XLSX)

S1 Fig Construction and characterization of weighted gene co-expression networks. (PDF)

S2 Fig Changes in choline metabolism–related genes and TLS-associated chemokine mRNA levels in NPC cells following SLC44A4 overexpression. (PDF)

S1 Protocol Supplemental materials and methods. (DOCX)

S1 File Raw images. (PDF)
