# Supplementary material for: Downregulation of SLC44A4 in nasopharyngeal carcinoma is associated with malignant progression, B-cell/TLS-related immune features, and sensitivity to DNA-damaging agents
Source: PLoS One. 2026 Jun 26;21(6):e0352812. doi: 10.1371/journal.pone.0352812 (PMC13308781; doi:10.1371/journal.pone.0352812)
Supplement: S1 Fig — (PDF) [file pone.0352812.s002.pdf]

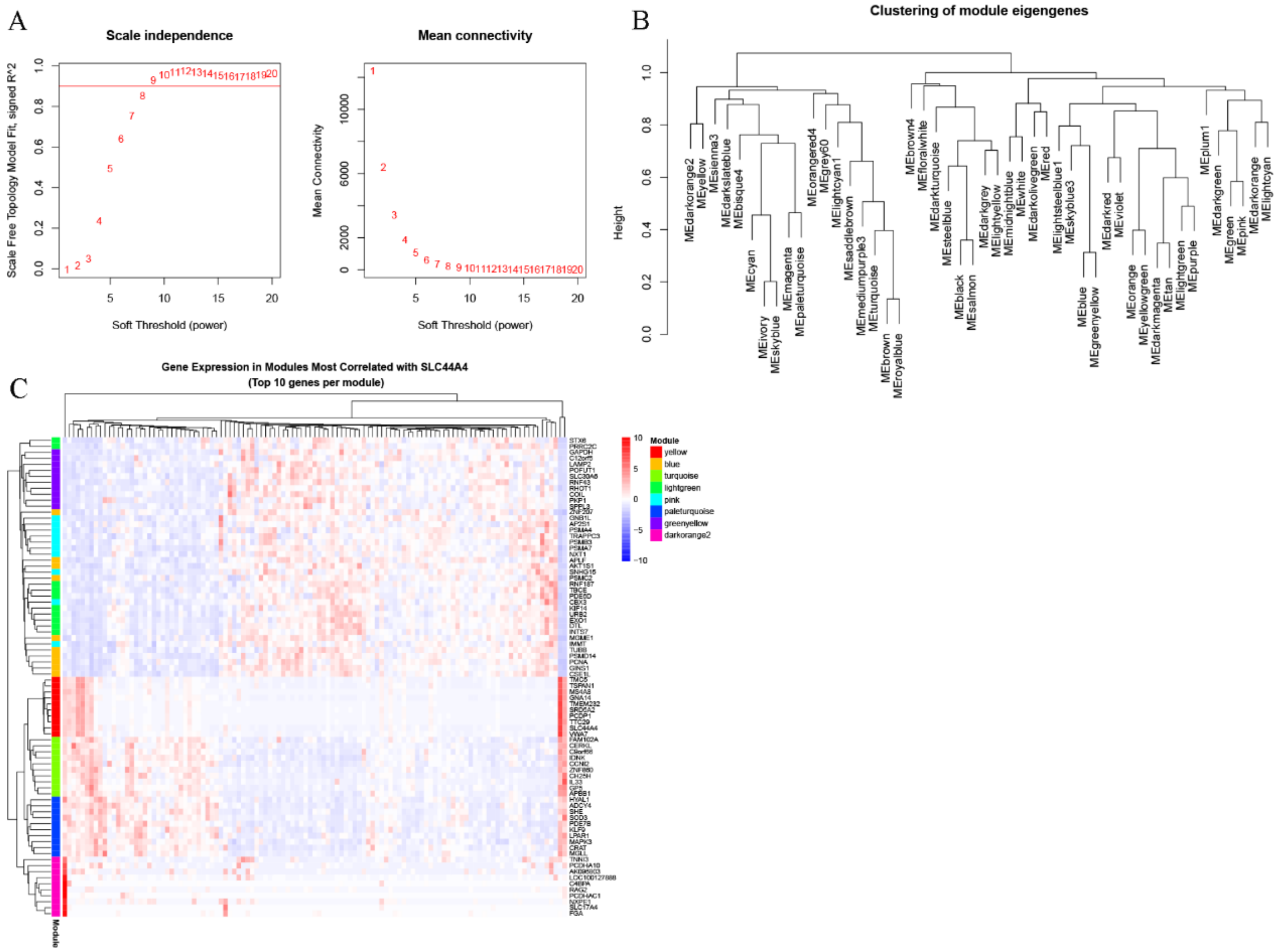

S1 Figure. Construction and characterization of weighted gene co-expression networks. (A) Network topology analysis for selection of the soft-thresholding power. The left panel shows the scale-free topology fit index as a function of the soft-thresholding power. The right panel depicts mean connectivity across different soft-thresholding powers; (B) Hierarchical clustering dendrogram of genes based on topological overlap, with branches representing distinct co-expression modules identified by WGCNA; (C) Gene expression heatmap of representative genes derived from modules most strongly correlated with SLC44A4 expression. For each selected module, the top 10 genes showing the highest correlation with SLC44A4 expression were visualized, illustrating coordinated expression patterns associated with SLC44A4.
