# Supplementary material for: Downregulation of SLC44A4 in nasopharyngeal carcinoma is associated with malignant progression, B-cell/TLS-related immune features, and sensitivity to DNA-damaging agents
Source: PLoS One. 2026 Jun 26;21(6):e0352812. doi: 10.1371/journal.pone.0352812 (PMC13308781; doi:10.1371/journal.pone.0352812)
Supplement: S2 Fig — (PDF) [file pone.0352812.s003.pdf]

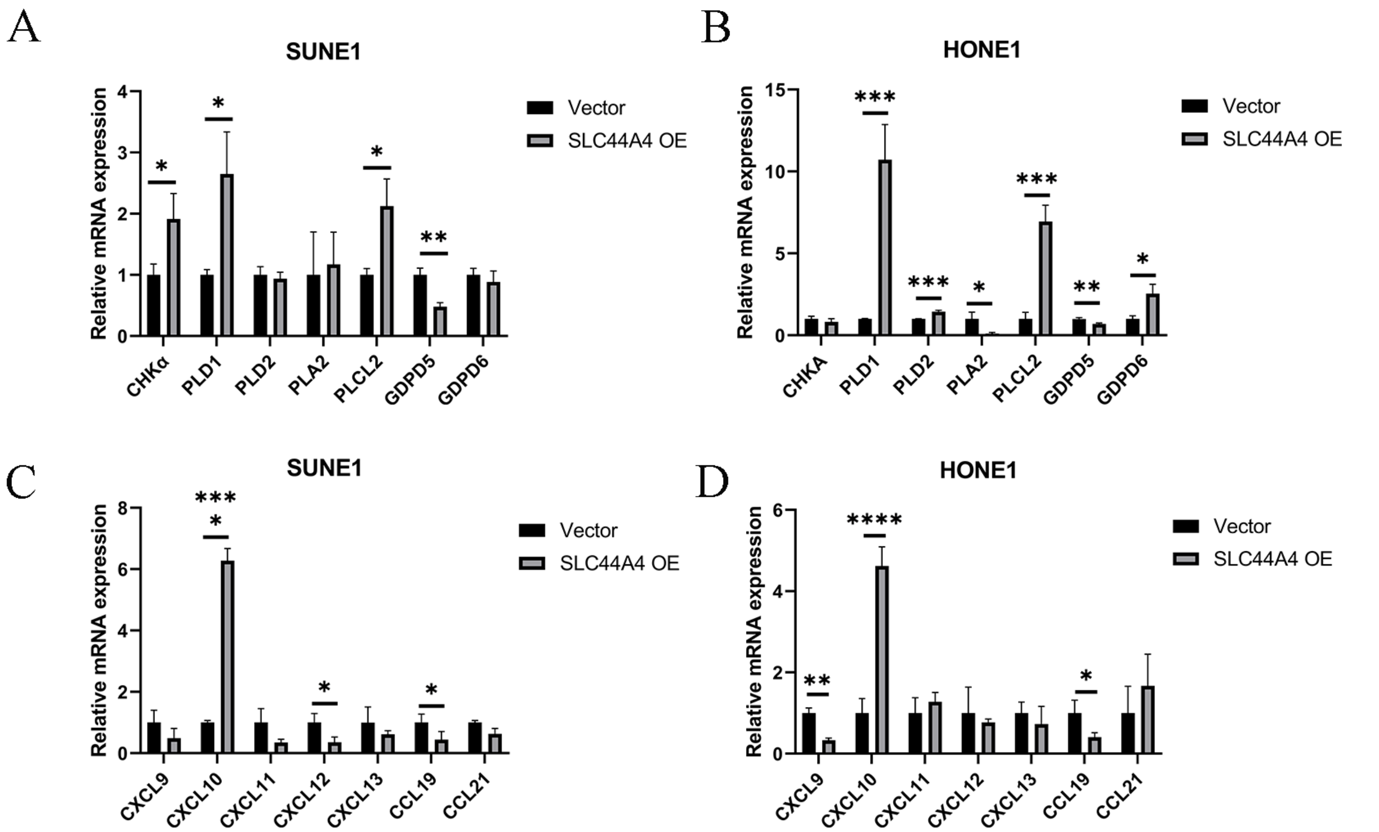


**S2 Figure. Changes in choline metabolism–related genes and TLS-associated chemokine mRNA levels in NPC cells following SLC44A4 overexpression.** (A–B) qPCR analysis showing that PLD1 and PLCL2 were the most consistently upregulated choline metabolism–related genes under SLC44A4 overexpression, whereas the other genes exhibited only modest or cell line–dependent changes. (C–D) SLC44A4 overexpression led to selective and significant upregulation of CXCL10 mRNA, a chemokine involved in inflammatory signaling and potentially associated with immune cell recruitment, while other chemokines did not show consistent changes across the two NPC cell lines.
