# Supplementary material for: Downregulation of SLC44A4 in nasopharyngeal carcinoma is associated with malignant progression, B-cell/TLS-related immune features, and sensitivity to DNA-damaging agents
Source: PLoS One. 2026 Jun 26;21(6):e0352812. doi: 10.1371/journal.pone.0352812 (PMC13308781; doi:10.1371/journal.pone.0352812)
Supplement: S1 Protocol — (DOCX) [file pone.0352812.s004.docx]

**Supplemental Materials and Methods**

**Drug sensitivity assay and IC50 determination**

All small-molecule drugs used in this study were purchased from MedChemExpress (MCE, USA), including cisplatin, doxorubicin, etoposide, temozolomide, olaparib, and 5-fluorouracil (5-FU). Stock solutions were prepared according to the manufacturer’s instructions (DMSO for most compounds; aqueous solution when applicable), aliquoted, and stored at −80 °C. For drug treatment, cells were seeded into 96-well plates at 2800-3200 cells per well and allowed to adhere overnight. Cells were then exposed to serially diluted concentrations of each drug (typically 5 concentrations with constant dilution ratio) for 48 h, with vehicle-treated cells serving as controls. The final concentration of DMSO was kept identical across groups and did not exceed 0.1% (v/v). Cell viability was measured using a Cell Counting Kit-8 (CCK-8) assay. Dose–response curves and half-maximal inhibitory concentrations (IC50) were calculated in GraphPad Prism 9 (GraphPad Software, USA) using nonlinear regression. Unless otherwise specified, each condition was tested in triplicate wells, and all experiments were independently repeated at least three times. Statistical analyses for drug-response experiments were performed based on IC50 values or normalized viability data as indicated in the figure legends.

**Primers and Plasmids**

The 2130 bp SLC44A4 Coding sequence (CDS) fragment was subcloned into the EcoR I / BamH I sites of the pLV3-CMV vector (Promega, Madison, WI, USA) and the plasmid was designated as pLV3-CMV-SLC44A4. The construct was verified by sequencing.

CXCL9-F CCAGTAGTGAGAAAGGGTCGC

CXCL9-R AGGGCTTGGGGCAAATTGTT

CXCL10-F GTGGCATTCAAGGAGTACCTC

CXCL10-R TGATGGCCTTCGATTCTGGATT

CXCL11-F GACGCTGTCTTTGCATAGGC

CXCL11-R GGATTTAGGCATCGTTGTCCTTT

CXCL12-F ATTCTCAACACTCCAAACTGTGC

CXCL12-R ACTTTAGCTTCGGGTCAATGC

CXCL13-F GCTTGAGGTGTAGATGTGTCC

CXCL13-R CCCACGGGGCAAGATTTGAA

CCL19-F TACATCGTGAGGAACTTCCACT

CCL19-R CTGGATGATGCGTTCTACCCA

CCL21-F GTTGCCTCAAGTACAGCCAAA

CCL21-R AGAACAGGATAGCTGGGATGG

CHKα-F GATCCGAACAAGCTCAGAAAGAAAATG

CHKα-R CGGCTCGGGATGAACTGCTC

PDL1-F GGAAAGCGTGACAGTGAAATG

PDL1-R GATAGCCAAGGACAACCCTAAA

PLD2-F TCGATTTGCCGTTGCCTATTC

PLD2-R GGTCAAGAGACGGTTGAGGTA

GDPD5-F CTACAACCCTGAGCAGAT

GDPD5-R AACATACGGAGAGCACAT

GDPD6-F TTTCAAAATGCTGCAGGGTAAT

GDPD6-R ACCCACAAAGCAACAGTGTGTA

PLA2-F CAGCTACCAGTTCCACATCGT

PLA2-R CGGATTGCTTGTCACACTCAC

PLCL2-F CAGGGTGTGGCACATATAAATGA

PLCL2-R GGACCTCGGAACTGATCCTCTA

**Flow cytometry analysis**

We used flow cytometry to analyze the cell cycle changes. Cells were seeded into six-well plates at a density of 5 × 105 cells per well. The cells were collected after 24 h of culture and then fixed with 70% ethanol. The fixed cells were stored at 4 °C overnight. The cells were rinsed with cold PBS after centrifugation (600×g, 5 min, 4 °C). Subse-quently, 500 μl PI/RNase (C1052, Beyotime, China), a dye working solution prepared in advance, was added to the cells to stain the DNA, and the cells were incubated for 30 min in the dark. Flow cytometry (GuavaeasyCyte, Merck, MA, USA) was used to analyze the stained cells, and Guava InCyte software was used to examine the percentage of cells present in the different cell cycle phases.

**Cell proliferation, plate colony-formation assays, migration and invasion**

In a 96-well plate, approximately 800–1200 cells were seeded per well. The CCK8 assay kit was used to measure absorbance values at 450 nm at 0 h, 24 h, 48 h, and 72 h to plot the proliferation curve. For the colony formation assay, approximately 800 cells per well were seeded in a 6-well plate. After visible colonies formed, the cells were fixed with methanol and stained with 0.1% crystal violet, followed by counting the number of colonies. For the cell migration assay, 5 × 10^4 cells per well were seeded in a 24-well Transwell plate. After cell migration, the upper chamber cells were removed, and the lower membrane-bound cells were fixed, stained, and counted. In the cell in-vasion assay, 80 μL of 10% Matrigel was added to the upper chamber of a 24-well Transwell plate, and 4×10^4 cells per well were seeded. After 36 hours of treatment, the upper chamber cells were removed, and the invasive cells on the lower membrane were fixed, stained, and quantified. All experiments were repeated at least thrice.

**EdU assay**

The EdU assay was performed using a EdU kit (C0081S, Beyotime). Briefly, after 3000 cells per well were seeded into 96-well plates, the EdU(5-ethynyl-2' -deoxyuridine) was employed to label proliferating cells. The number of cells labeled with EdU was then calculated.

**Western blotting**

Cells were harvested and washed three times with PBS, then disrupted in IP lysis buffer (25 mM Tris-HCl, pH 7.4, 150 mM NaCl, 1% NP40, 1 mM EDTA, 5% glycerol; Thermo Scientific, MA, USA). Extracted proteins were quantified and separated by SDS-PAGE and transferred onto PVDF membranes. Before incubation with the primary antibody, the PVDF membrane was cut based on the size of the target molecules, retaining the bands around 36kDa for incubation with the GAPDH antibody and approximately 79kDa for SLC44A4 antibody incubation. After overnight incubation with the primary antibody, peroxidase-conjugated secondary antibodies were used to detect binding of primary antibodies. Visualization was performed by using the ChemiDoc XRS system with Image Lab software (Bio-Rad, CA, USA). The primary antibodies used were GAPDH (Cat#60004-1-lg, Proteintech, Wuhan, China), SLC44A4 (Cat#HA600017, HUABIO, Hangzhou, China) and OXPHOS Cocktail (Cat# PK30006, Proteintech, Wuhan, China).

**Hematoxylin and Eosin (H&E) and Immunohistochemistry (IHC) staining**

Samples of tumor tissue were fixed at least 24 h in 4% paraformaldehyde solution. The tissues were sliced into 6mm thick sections after gradient dehydration and paraffin embedding, deparaffinized in xylene, and rehydrated using a gradient of ethanol so-lutions with progressively lower concentrations. The sections were utilized for H&E staining to assess alterations in tissue structure. Primary antibody against SLC44A4 was used to conduct IHC analysis of the tissue samples. Staining results were evaluated and photographed using a microscope (Leica, Germany).
