# Supplementary figures and images for: Downregulation of SLC44A4 in nasopharyngeal carcinoma is associated with malignant progression, B-cell/TLS-related immune features, and sensitivity to DNA-damaging agents
Source: PLoS One. 2026 Jun 26;21(6):e0352812. doi: 10.1371/journal.pone.0352812 (PMC13308781; doi:10.1371/journal.pone.0352812)

Row image

Fig. 2H

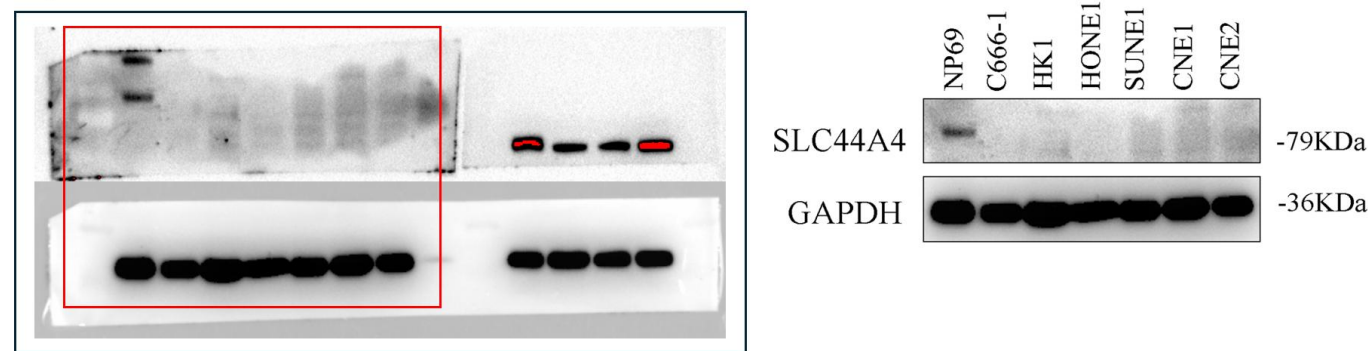

Fig. 5A

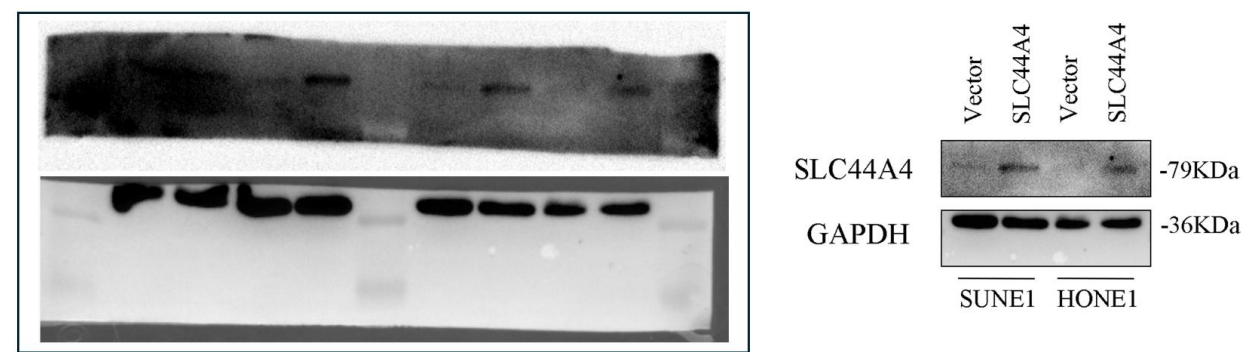

Fig. 6B

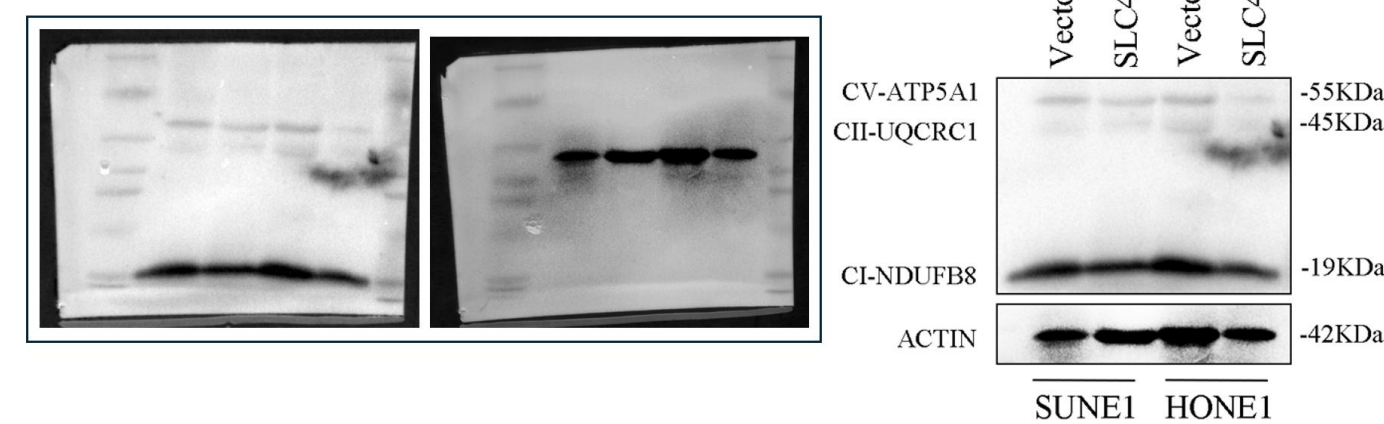

Supplement: S1 File — (PDF) [file pone.0352812.s005.pdf]
