# Supplementary material for: Downregulation of SLC44A4 in nasopharyngeal carcinoma is associated with malignant progression, B-cell/TLS-related immune features, and sensitivity to DNA-damaging agents
Source: PLoS One. 2026 Jun 26;21(6):e0352812. doi: 10.1371/journal.pone.0352812 (PMC13308781; doi:10.1371/journal.pone.0352812)
Supplement: Attachment — Submitted filename: Point-by-Point Response.docx [file pone.0352812.s007.docx]

**Point-by-Point Response**

**Reviewer #1:** The manuscript by Liu et al. revealed the multiple functions of SLC44A4 as a tumor suppressor in nasopharyngeal carcinoma. By integrating single-cell sequencing, functional experiments and bioinformatics analysis, the authors discovered that SLC44A4 not only inhibits tumor proliferation and invasion, but also enhances DNA damage drug sensitivity and promotes B-cell infiltration and tertiary lymphoid structure formation, providing a new perspective for understanding the complex role of transporters in tumors. However, some minor concerns should be addressed to further improve the current version of the manuscript for its publication in this journal.

**Response:** Thank you for your careful review and positive comments! We are also grateful for your constructive suggestions, which we believe will significantly improve the clarity and impact of our manuscript.

**Comment 1:** The conclusion that jumps directly from "DNA damage sensitivity" to "therapeutic target" is rather hasty and does not clarify whether it is based on SLC44A4 as the drug target itself or as a biomarker for predicting chemotherapy sensitivity.

**Response:** We thank the reviewer for raising this important point. We agree that the previous wording may have overextended the implication from sensitivity to DNA-damaging agents to “therapeutic target” without sufficiently distinguishing whether SLC44A4 itself is a druggable target or a predictive biomarker.

In the revised manuscript, we have clarified this point. Based on the current data, SLC44A4 should be interpreted primarily as a potential biomarker for predicting sensitivity to DNA damage-based therapies, rather than as a directly validated therapeutic target.

**Comment 2:** What is the potential molecular mechanism by which SLC44A4 regulates DNA damage responses, such as influencing membrane repair by regulating choline metabolism? Or by influencing ROS levels?

**Response:** We appreciate the reviewer's insightful question. Our previous bioinformatics analyses showed that SLC44A4 expression was significantly negatively correlated with OXPHOS-related signatures, suggesting a potential link between SLC44A4 and mitochondrial metabolic remodeling. Given that mitochondrial OXPHOS is closely associated with cellular redox homeostasis, ROS generation, and the energetic demands of DNA damage repair processes (PMID: 38081963, PMID: 41310084), we performed additional Western blot analyses to validate this association. We found that SLC44A4 overexpression markedly reduced the protein levels of NDUFB8, UQCRC1, and ATP5A1, representative subunits of mitochondrial complexes I, III, and V, respectively (Fig.6B). These findings suggest that SLC44A4 overexpression is associated with mitochondrial OXPHOS remodeling in NPC cells. Given that mitochondrial respiratory activity can influence cellular redox homeostasis, metabolic stress adaptation, and responses to DNA damage-based therapies, these results provide a plausible mechanistic link between SLC44A4 and enhanced drug sensitivity. However, we agree that the current data do not directly establish ROS-dependent or DNA repair-dependent mechanisms. We have therefore revised the manuscript to present this pathway as a hypothesis and added this issue as a limitation requiring further validation by ROS measurement, mitochondrial respiration assays, and DNA damage/repair kinetics analyses. The corresponding changes have been made in the revised manuscript on Page 11, Lines 269–272 and Page 13, Lines 343–353.

**Figure 6B**

**Comment 3:** The logical connection between the analysis of the immune microenvironment and the intrinsic functions of tumor cells is not close enough.

**Response:** We thank the reviewer for raising this important point. In the revised manuscript, we have strengthened the logical connection between these two sections. We have revised the wording to avoid implying that SLC44A4 directly induces TLS formation. Instead, we now describe this finding as an association between SLC44A4 expression and a TLS/B cell-enriched immune phenotype. In addition, we found that SLC44A4 overexpression significantly increased the mRNA expression of CXCL10 in NPC cells. We have also added a discussion of CXCL10 as a possible mediator, while clearly acknowledging that the causal relationship remains to be experimentally established. The corresponding changes have been made in the revised manuscript on Page 11, Lines 273–277 and Page 14, Lines 365–376.

**Comment 4:** To further evaluate the feasibility of SLC44A4 expression levels guiding individualized treatment strategies could enhance the clinical significance of this research.

**Response:** We thank the reviewer for this valuable suggestion.

In the revised Discussion, we have added a section addressing the potential clinical application of SLC44A4:

“From a translational medicine perspective, SLC44A4 expression may have potential value in biomarker-guided therapeutic strategies. Our data raise the possibility that patients with high SLC44A4 expression may be more responsive to DNA damage–targeting treatments, such as platinum-based chemotherapy, radiotherapy, or DNA damage–based combination strategies but may derive less benefit from fluoropyrimidine-based treatment such as 5-FU. In addition, the association between SLC44A4 expression and B-cell/TLS-related immune features suggests that this gene may be relevant to immune microenvironment-based stratification. However, these possibilities require further validation. Prospective clinical cohorts incorporating treatment response data, survival outcomes, standardized methods for SLC44A4 detection, and predefined cutoff values will be necessary before SLC44A4 can be applied to guide individualized treatment in clinical practice.”

**Comment 5:** The abstract is too simple, did not include some prominence results and important conclusions.

**Response:** We thank the reviewer for this helpful comment. In the revised manuscript, we have expanded the Abstract to better summarize the key results and conclusions:

“Nasopharyngeal carcinoma (NPC) is characterized by aggressive progression, frequent metastasis, and heterogeneous therapeutic responses. Through integrated bulk transcriptomic datasets, single-cell analysis, clinical specimens, and NPC cell lines, we identified SLC44A4, a member of the choline transporter-like family, as a significantly downregulated gene in NPC. Transcriptomic analyses revealed that SLC44A4 expression was negatively associated with oxidative phosphorylation–related genes, DNA damage repair pathways, and malignant transcriptional programs related to proliferation, invasion, and metastasis. Moreover, SLC44A4-high tumors exhibited increased B-cell infiltration and enrichment of TLS-related transcriptional signatures. In vitro, SLC44A4 overexpression in NPC cells suppressed proliferation, colony formation, migration, and invasion, induced G0/G1 cell-cycle arrest, reduced expression of oxidative phosphorylation–related proteins, and selectively upregulated CXCL10. SLC44A4 overexpression also increased sensitivity to DNA-damaging agents, including temozolomide, doxorubicin, cisplatin, olaparib, and etoposide, while decreasing sensitivity to 5-fluorouracil.

Together, these findings identify SLC44A4 as a potential tumor-suppressive factor in NPC and suggest that SLC44A4 may serve as a biomarker for metabolic state, B-cell/TLS-associated immune features, and vulnerability to DNA damage–based therapies.”

**Comment 6:** There are some grammatical errors in the text Suggest the authors carefully check throughout the manuscript and revise them.

**Response:** We thank the reviewer for pointing this out. We have carefully checked the entire manuscript and revised grammatical errors, awkward expressions, and unclear sentences. We also improved the consistency of terminology throughout the manuscript.

**Comment 7:** Some references are outdated and the formats are not uniform.

**Response:** We thank the reviewer for this helpful suggestion. We have carefully reviewed the reference list, updated relevant citations where appropriate, and standardized the reference format according to the journal requirements.

**Reviewer #2:** The study aims to investigate the expression, clinical significance, and functional role of solute carrier family 44 member 4 (SLC44A4) in NPC. It specifically explores the link between this transporter and tumor suppression, DNA damage vulnerability, and the immune microenvironment. Minor recommendations for the author.

**Response:** We sincerely thank the reviewer for the positive evaluation of our study and for the constructive suggestions. In response to the reviewer’s minor recommendations, we have carefully revised and improved the manuscript point by point. In the revised manuscript, we have further clarified the relevant statements and added necessary explanations to enhance the accuracy and readability of the paper.

**Comment 1:** Validation of choline transport activity:

The authors acknowledge that while the SLC44 family is implicated in choline transport, SLC44A4's specific activity remains unproven. Experimental confirmation of its transport function would solidify the link to the "Choline metabolism in cancer" pathway.

**Response:** We thank the reviewer for this important suggestion. In the revised study, we performed additional qPCR analyses to examine whether SLC44A4 overexpression affects genes involved in choline/phospholipid metabolism (Figure S2). We found that PLD1 and PLCL2 were consistently upregulated in both HONE1 and SUNE1 cells following SLC44A4 overexpression, whereas other examined genes showed limited or cell line-dependent changes. These results suggest that SLC44A4 may be associated with remodeling of choline/phospholipid metabolism-related transcriptional programs in NPC cells.

However, we acknowledge that these data do not directly demonstrate choline transport activity of SLC44A4. Therefore, we have revised the manuscript to avoid overstatement and now describe SLC44A4 as being associated with choline/phospholipid metabolic remodeling rather than as a confirmed choline transporter in NPC. We have also added this point as a limitation and indicated that future studies using radiolabeled or isotope-labeled choline uptake assays, choline metabolic flux analysis, and lipidomic profiling are required to determine whether SLC44A4 directly mediates choline transport.

**Figure S2**

**Comment 2:** Elucidate TLS Recruitment Mechanism:
While the authors successfully demonstrate a strong correlation between SLC44A4 and TLS/B-cell infiltration, the underlying molecular mechanism remains unclear. Specifically, it is not yet defined which chemokines are secreted by SLC44A4 to drive this process. Identifying these key molecular mediators would significantly elevate the mechanistic depth of the work and better support its clinical relevance.

**Response:** We thank the reviewer for raising this important mechanistic question. To explore whether SLC44A4 alters the expression of chemokine genes involved in TLS formation and B-cell recruitment, we performed additional qPCR analyses in SLC44A4-overexpressing NPC cells. We examined several TLS- and immune recruitment-related chemokines, including CXCL13, CCL19, CCL21, CXCL12, CXCL9, CXCL10, and CXCL11. We found that SLC44A4 overexpression significantly increased the mRNA expression of CXCL10, which is also one of the major chemokines secreted by tumor cells. In contrast, CXCL13, CCL19, CCL21 and CXCL12, which are typical TLS/B cell-associated chemokines, did not show consistent upregulation.

These data suggest that SLC44A4 overexpression does not directly activate the classical TLS-organizing chemokine program in NPC tumor cells. Instead, SLC44A4 may be associated with a more inflammatory or immune-permissive tumor-cell state. The consistent upregulation of CXCL10 suggests a potential link with the CXCL10/CXCR3 axis, which may facilitate recruitment or functional positioning of CXCR3⁺ immune cells in certain inflammatory and tumor contexts (PMID: 37699392, PMID: 40009768, PMID: 41617729). However, CXCL10 alone is unlikely to be sufficient to organize TLS formation. Mature TLS development generally requires coordinated lymphoid-organizing modules, including CXCL13–CXCR5, CCL19/CCL21–CCR7, LTβR–NF-κB signaling, HEVs, and FDC-associated stromal programs (PMID: 39117919). CXCL10 is therefore more likely to function as an amplifier of TLS functionalization/inflammation rather than as an autonomous TLS-organizing factor. We have revised the manuscript accordingly to avoid implying a direct causal role of SLC44A4 in TLS formation and have added this point to the Discussion. The corresponding changes have been made in the revised manuscript on Page 11, Lines 273–277 and Page 14, Lines 365–376.

**Figure S2**
