# Supplementary material for: Downregulation of SLC44A4 in nasopharyngeal carcinoma is associated with malignant progression, B-cell/TLS-related immune features, and sensitivity to DNA-damaging agents
Source: PLoS One. 2026 Jun 26;21(6):e0352812. doi: 10.1371/journal.pone.0352812 (PMC13308781; doi:10.1371/journal.pone.0352812)
Supplement: Peer review history

# Peer review: Decision Letter 0

**DOI:** 10.1371/journal.pone.0352812.r001

**Authors:** Peng Zhang

5 May 2026

PONE-D-26-10507

SLC44A4 in Nasopharyngeal Carcinoma: A Novel Prognostic Biomarker Linking Tumor Suppression, DNA Damage Vulnerability, and Tertiary Lymphoid Structure–Mediated Immunity

PLOS One

Dear Dr. Liu,

Thank you for submitting your manuscript to PLOS ONE. After careful consideration, we feel that it has merit but does not fully meet PLOS ONE’s publication criteria as it currently stands. Therefore, we invite you to submit a revised version of the manuscript that addresses the points raised during the review process.

Please submit your revised manuscript by Jun 19 2026 11:59PM. If you will need more time than this to complete your revisions, please reply to this message or contact the journal office at plosone@plos.org. When you're ready to submit your revision, log on to https://www.editorialmanager.com/pone/ and select the 'Submissions Needing Revision' folder to locate your manuscript file.

Please include the following items when submitting your revised manuscript:

- A letter that responds to each point raised by the academic editor and reviewer(s). You should upload this letter as a separate file labeled 'Response to Reviewers'.
- A marked-up copy of your manuscript that highlights changes made to the original version. You should upload this as a separate file labeled 'Revised Manuscript with Track Changes'.
- An unmarked version of your revised paper without tracked changes. You should upload this as a separate file labeled 'Manuscript'.

If you would like to make changes to your financial disclosure, please include your updated statement in your cover letter. Guidelines for resubmitting your figure files are available below the reviewer comments at the end of this letter.

If applicable, we recommend that you deposit your laboratory protocols in protocols.io to enhance the reproducibility of your results. Protocols.io assigns your protocol its own identifier (DOI) so that it can be cited independently in the future. For instructions see: https://journals.plos.org/plosone/s/submission-guidelines#loc-laboratory-protocols. Additionally, PLOS ONE offers an option for publishing peer-reviewed Lab Protocol articles, which describe protocols hosted on protocols.io. Read more information on sharing protocols at https://plos.org/protocols?utm_medium=editorial-email&utm_source=authorletters&utm_campaign=protocols.

As the corresponding author, your ORCID iD is verified in the submission system and will appear in the published article. PLOS supports the use of ORCID, and we encourage all coauthors to register for an ORCID iD and use it as well. Please encourage your coauthors to verify their ORCID iD within the submission system before final acceptance, as unverified ORCID iDs will not appear in the published article. *Only*  the individual author can complete the verification step; PLOS staff *cannot*  verify ORCID iDs on behalf of authors.

We look forward to receiving your revised manuscript.

Kind regards,

Peng Zhang, Ph.D.

Academic Editor

PLOS One

Journal Requirements:

When submitting your revision, we need you to address these additional requirements.

1. Please ensure that your manuscript meets PLOS ONE's style requirements, including those for file naming. The PLOS ONE style templates can be found at https://journals.plos.org/plosone/s/file?id=wjVg/PLOSOne_formatting_sample_main_body.pdf and https://journals.plos.org/plosone/s/file?id=ba62/PLOSOne_formatting_sample_title_authors_affiliations.pdf

2. Please note that PLOS One has specific guidelines on code sharing for submissions in which author-generated code underpins the findings in the manuscript. In these cases, we expect all author-generated code to be made available without restrictions upon publication of the work. Please review our guidelines at https://journals.plos.org/plosone/s/materials-and-software-sharing#loc-sharing-code and ensure that your code is shared in a way that follows best practice and facilitates reproducibility and reuse.

3. We note that the grant information you provided in the ‘Funding Information’ and ‘Financial Disclosure’ sections do not match.

When you resubmit, please ensure that you provide the correct grant numbers for the awards you received for your study in the ‘Funding Information’ section.

4. Thank you for stating the following financial disclosure:

[The author(s) received no specific funding for this work.].

At this time, please address the following queries:

a) Please clarify the sources of funding (financial or material support) for your study. List the grants or organizations that supported your study, including funding received from your institution.

b) State what role the funders took in the study. If the funders had no role in your study, please state: “The funders had no role in study design, data collection and analysis, decision to publish, or preparation of the manuscript.”

c) If any authors received a salary from any of your funders, please state which authors and which funders.

d) If you did not receive any funding for this study, please state: “The authors received no specific funding for this work.”

Please include your amended statements within your cover letter; we will change the online submission form on your behalf.

5. Thank you for stating the following in your manuscript:

[This work was supported by grants from the Fundamental Research Funds for the Central Universities of Central South University (2025ZZTS0140) and Hunan Province Graduate Research Innovation Project (CX20250336).]

We note that you have provided funding information that is not currently declared in your Funding Statement. However, funding information should not appear in the Acknowledgments section or other areas of your manuscript. We will only publish funding information present in the Funding Statement section of the online submission form.

Please remove any funding-related text from the manuscript and let us know how you would like to update your Funding Statement. Currently, your Funding Statement reads as follows:

[The author(s) received no specific funding for this work.]

Please include your amended statements within your cover letter; we will change the online submission form on your behalf.

6. PLOS ONE now requires that authors provide the original uncropped and unadjusted images underlying all blot or gel results reported in a submission’s figures or Supporting Information files. This policy and the journal’s other requirements for blot/gel reporting and figure preparation are described in detail at https://journals.plos.org/plosone/s/figures#loc-blot-and-gel-reporting-requirements and https://journals.plos.org/plosone/s/figures#loc-preparing-figures-from-image-files. When you submit your revised manuscript, please ensure that your figures adhere fully to these guidelines and provide the original underlying images for all blot or gel data reported in your submission. See the following link for instructions on providing the original image data: https://journals.plos.org/plosone/s/figures#loc-original-images-for-blots-and-gels

In your cover letter, please note whether your blot/gel image data are in Supporting Information or posted at a public data repository, provide the repository URL if relevant, and provide specific details as to which raw blot/gel images, if any, are not available. Email us at plosone@plos.org if you have any questions.

7. Thank you for stating the following in the Competing Interests section:

[The authors have declared that no competing interests exist.].

We note that one or more of the authors are employed by a commercial company: Changsha Medical University.

1. Please provide an amended Funding Statement declaring this commercial affiliation, as well as a statement regarding the Role of Funders in your study. If the funding organization did not play a role in the study design, data collection and analysis, decision to publish, or preparation of the manuscript and only provided financial support in the form of authors' salaries and/or research materials, please review your statements relating to the author contributions, and ensure you have specifically and accurately indicated the role(s) that these authors had in your study. You can update author roles in the Author Contributions section of the online submission form.

Please also include the following statement within your amended Funding Statement.

“The funder provided support in the form of salaries for authors [insert relevant initials], but did not have any additional role in the study design, data collection and analysis, decision to publish, or preparation of the manuscript. The specific roles of these authors are articulated in the ‘author contributions’ section.”

If your commercial affiliation did play a role in your study, please state and explain this role within your updated Funding Statement.

2. Please also provide an updated Competing Interests Statement declaring this commercial affiliation along with any other relevant declarations relating to employment, consultancy, patents, products in development, or marketed products, etc.

Within your Competing Interests Statement, please confirm that this commercial affiliation does not alter your adherence to all PLOS ONE policies on sharing data and materials by including the following statement: "This does not alter our adherence to PLOS ONE policies on sharing data and materials.” (as detailed online in our guide for authors http://journals.plos.org/plosone/s/competing-interests) . If this adherence statement is not accurate and there are restrictions on sharing of data and/or materials, please state these. Please note that we cannot proceed with consideration of your article until this information has been declared.

Please include both an updated Funding Statement and Competing Interests Statement in your cover letter. We will change the online submission form on your behalf.

8. Please upload a new copy of Figure 1 as the detail is not clear. Please follow the link for more information:  https://journals.plos.org/plosone/s/figures

9. Please include captions for your Supporting Information files at the end of your manuscript, and update any in-text citations to match accordingly. Please see our Supporting Information guidelines for more information: http://journals.plos.org/plosone/s/supporting-information.

If the reviewer comments include a recommendation to cite specific previously published works, please review and evaluate these publications to determine whether they are relevant and should be cited. There is no requirement to cite these works unless the editor has indicated otherwise.

Please review your reference list to ensure that it is complete and correct. If you have cited papers that have been retracted, please include the rationale for doing so in the manuscript text, or remove these references and replace them with relevant current references. Any changes to the reference list should be mentioned in the rebuttal letter that accompanies your revised manuscript. If you need to cite a retracted article, indicate the article’s retracted status in the References list and also include a citation and full reference for the retraction notice.

[Note: HTML markup is below. Please do not edit.]

Reviewers' comments:

Reviewer's Responses to Questions

**Comments to the Author**

1. Is the manuscript technically sound, and do the data support the conclusions?

The manuscript must describe a technically sound piece of scientific research with data that supports the conclusions. Experiments must have been conducted rigorously, with appropriate controls, replication, and sample sizes. The conclusions must be drawn appropriately based on the data presented.

Reviewer #1: Yes

Reviewer #2: Yes

**********

2. Has the statistical analysis been performed appropriately and rigorously?

Reviewer #1: I Don't Know

Reviewer #2: I Don't Know

**********

3. Have the authors made all data underlying the findings in their manuscript fully available?

The PLOS Data policy requires authors to make all data underlying the findings described in their manuscript fully available without restriction, with rare exception (please refer to the Data Availability Statement in the manuscript PDF file). The data should be provided as part of the manuscript or its supporting information, or deposited to a public repository. For example, in addition to summary statistics, the data points behind means, medians and variance measures should be available. If there are restrictions on publicly sharing data—e.g. participant privacy or use of data from a third party—those must be specified.

Reviewer #1: Yes

Reviewer #2: Yes

**********

4. Is the manuscript presented in an intelligible fashion and written in standard English?

PLOS ONE does not copyedit accepted manuscripts, so the language in submitted articles must be clear, correct, and unambiguous. Any typographical or grammatical errors should be corrected at revision, so please note any specific errors here.

Reviewer #1: Yes

Reviewer #2: Yes

**********

5. Review Comments to the Author

Please use the space provided to explain your answers to the questions above. You may also include additional comments for the author, including concerns about dual publication, research ethics, or publication ethics. (Please upload your review as an attachment if it exceeds 20,000 characters)

Reviewer #1: The manuscript by Liu et al. revealed the multiple functions of SLC44A4 as a tumor suppressor in nasopharyngeal carcinoma. By integrating single-cell sequencing, functional experiments and bioinformatics analysis, the authors discovered that SLC44A4 not only inhibits tumor proliferation and invasion, but also enhances DNA damage drug sensitivity and promotes B-cell infiltration and tertiary lymphoid structure formation, providing a new perspective for understanding the complex role of transporters in tumors. However, some minor concerns should be addressed to further improve the current version of the manuscript for its publication in this journal.

1.The conclusion that jumps directly from "DNA damage sensitivity "to" therapeutic target "is rather hasty and does not clarify whether it is based on SLC44A4 as the drug target itself or as a biomarker for predicting chemotherapy sensitivity;

2.What is the potential molecular mechanism by which SLC44A4 regulates DNA damage responses, such as influencing membrane repair by regulating choline metabolism? Or by influencing ROS levels?

3.The logical connection between the analysis of the immune microenvironment and the intrinsic functions of tumor cells is not close enough;

4.To further evaluate the feasibility of SLC44A4 expression levels guiding individualized treatment strategies could enhance the clinical significance of this research.

5.The abstract is too simple, did not include some prominence results and important conclusions.

6.There are some grammatical errors in the text！ Suggest the authors carefully check throughout the manuscript and revise them.

7. Some references are outdated and the formats are not uniform.

Reviewer #2: The study aims to investigate the expression, clinical significance, and functional role of solute carrier family 44 member 4 (SLC44A4) in NPC. It specifically explores the link between this transporter and tumor suppression, DNA damage vulnerability, and the immune microenvironment.

Minor recommendations for the author:

1. Validation of choline transport activity:

The authors acknowledge that while the SLC44 family is implicated in choline transport, SLC44A4's specific activity remains unproven. Experimental confirmation of its transport function would solidify the link to the "Choline metabolism in cancer" pathway.

2. Elucidate TLS Recruitment Mechanism:

While the authors successfully demonstrate a strong correlation between SLC44A4 and TLS/B-cell infiltration, the underlying molecular mechanism remains unclear. Specifically, it is not yet defined which chemokines are secreted by SLC44A4 to drive this process. Identifying these key molecular mediators would significantly elevate the mechanistic depth of the work and better support its clinical relevance.

**********

6. PLOS authors have the option to publish the peer review history of their article (what does this mean?). If published, this will include your full peer review and any attached files.

If you choose “no”, your identity will remain anonymous but your review may still be made public.

**Do you want your identity to be public for this peer review?**  For information about this choice, including consent withdrawal, please see our Privacy Policy.

Reviewer #1: No

Reviewer #2: No

**********

[NOTE: If reviewer comments were submitted as an attachment file, they will be attached to this email and accessible via the submission site. Please log into your account, locate the manuscript record, and check for the action link "View Attachments". If this link does not appear, there are no attachment files.]

To ensure your figures meet our technical requirements, please review our figure guidelines: https://journals.plos.org/plosone/s/figures

You may also use PLOS’s free figure tool, NAAS, to help you prepare publication quality figures: https://journals.plos.org/plosone/s/figures#loc-tools-for-figure-preparation.

NAAS will assess whether your figures meet our technical requirements by comparing each figure against our figure specifications.

# Author comment: Author response to Decision Letter 1

**DOI:** 10.1371/journal.pone.0352812.r002

13 May 2026

Point-by-Point Response

Reviewer #1: The manuscript by Liu et al. revealed the multiple functions of SLC44A4 as a tumor suppressor in nasopharyngeal carcinoma. By integrating single-cell sequencing, functional experiments and bioinformatics analysis, the authors discovered that SLC44A4 not only inhibits tumor proliferation and invasion, but also enhances DNA damage drug sensitivity and promotes B-cell infiltration and tertiary lymphoid structure formation, providing a new perspective for understanding the complex role of transporters in tumors. However, some minor concerns should be addressed to further improve the current version of the manuscript for its publication in this journal.

Response: Thank you for your careful review and positive comments! We are also grateful for your constructive suggestions, which we believe will significantly improve the clarity and impact of our manuscript.

Comment 1: The conclusion that jumps directly from "DNA damage sensitivity" to "therapeutic target" is rather hasty and does not clarify whether it is based on SLC44A4 as the drug target itself or as a biomarker for predicting chemotherapy sensitivity.

Response: We thank the reviewer for raising this important point. We agree that the previous wording may have overextended the implication from sensitivity to DNA-damaging agents to “therapeutic target” without sufficiently distinguishing whether SLC44A4 itself is a druggable target or a predictive biomarker.

In the revised manuscript, we have clarified this point. Based on the current data, SLC44A4 should be interpreted primarily as a potential biomarker for predicting sensitivity to DNA damage-based therapies, rather than as a directly validated therapeutic target.

Comment 2: What is the potential molecular mechanism by which SLC44A4 regulates DNA damage responses, such as influencing membrane repair by regulating choline metabolism? Or by influencing ROS levels?

Response: We appreciate the reviewer's insightful question. Our previous bioinformatics analyses showed that SLC44A4 expression was significantly negatively correlated with OXPHOS-related signatures, suggesting a potential link between SLC44A4 and mitochondrial metabolic remodeling. Given that mitochondrial OXPHOS is closely associated with cellular redox homeostasis, ROS generation, and the energetic demands of DNA damage repair processes (PMID: 38081963, PMID: 41310084), we performed additional Western blot analyses to validate this association. We found that SLC44A4 overexpression markedly reduced the protein levels of NDUFB8, UQCRC1, and ATP5A1, representative subunits of mitochondrial complexes I, III, and V, respectively (Fig.6B). These findings suggest that SLC44A4 overexpression is associated with mitochondrial OXPHOS remodeling in NPC cells. Given that mitochondrial respiratory activity can influence cellular redox homeostasis, metabolic stress adaptation, and responses to DNA damage-based therapies, these results provide a plausible mechanistic link between SLC44A4 and enhanced drug sensitivity. However, we agree that the current data do not directly establish ROS-dependent or DNA repair-dependent mechanisms. We have therefore revised the manuscript to present this pathway as a hypothesis and added this issue as a limitation requiring further validation by ROS measurement, mitochondrial respiration assays, and DNA damage/repair kinetics analyses. The corresponding changes have been made in the revised manuscript on Page 11, Lines 269–272 and Page 13, Lines 343–353.

Comment 3: The logical connection between the analysis of the immune microenvironment and the intrinsic functions of tumor cells is not close enough.

Response: We thank the reviewer for raising this important point. In the revised manuscript, we have strengthened the logical connection between these two sections. We have revised the wording to avoid implying that SLC44A4 directly induces TLS formation. Instead, we now describe this finding as an association between SLC44A4 expression and a TLS/B cell-enriched immune phenotype. In addition, we found that SLC44A4 overexpression significantly increased the mRNA expression of CXCL10 in NPC cells. We have also added a discussion of CXCL10 as a possible mediator, while clearly acknowledging that the causal relationship remains to be experimentally established. The corresponding changes have been made in the revised manuscript on Page 11, Lines 273–277 and Page 14, Lines 365–376.

Comment 4: To further evaluate the feasibility of SLC44A4 expression levels guiding individualized treatment strategies could enhance the clinical significance of this research.

Response: We thank the reviewer for this valuable suggestion.

In the revised Discussion, we have added a section addressing the potential clinical application of SLC44A4:

“From a translational medicine perspective, SLC44A4 expression may have potential value in biomarker-guided therapeutic strategies. Our data raise the possibility that patients with high SLC44A4 expression may be more responsive to DNA damage–targeting treatments, such as platinum-based chemotherapy, radiotherapy, or DNA damage–based combination strategies but may derive less benefit from fluoropyrimidine-based treatment such as 5-FU. In addition, the association between SLC44A4 expression and B-cell/TLS-related immune features suggests that this gene may be relevant to immune microenvironment-based stratification. However, these possibilities require further validation. Prospective clinical cohorts incorporating treatment response data, survival outcomes, standardized methods for SLC44A4 detection, and predefined cutoff values will be necessary before SLC44A4 can be applied to guide individualized treatment in clinical practice.”

Comment 5: The abstract is too simple, did not include some prominence results and important conclusions.

Response: We thank the reviewer for this helpful comment. In the revised manuscript, we have expanded the Abstract to better summarize the key results and conclusions:

“Nasopharyngeal carcinoma (NPC) is characterized by aggressive progression, frequent metastasis, and heterogeneous therapeutic responses. Through integrated bulk transcriptomic datasets, single-cell analysis, clinical specimens, and NPC cell lines, we identified SLC44A4, a member of the choline transporter-like family, as a significantly downregulated gene in NPC. Transcriptomic analyses revealed that SLC44A4 expression was negatively associated with oxidative phosphorylation–related genes, DNA damage repair pathways, and malignant transcriptional programs related to proliferation, invasion, and metastasis. Moreover, SLC44A4-high tumors exhibited increased B-cell infiltration and enrichment of TLS-related transcriptional signatures. In vitro, SLC44A4 overexpression in NPC cells suppressed proliferation, colony formation, migration, and invasion, induced G0/G1 cell-cycle arrest, reduced expression of oxidative phosphorylation–related proteins, and selectively upregulated CXCL10. SLC44A4 overexpression also increased sensitivity to DNA-damaging agents, including temozolomide, doxorubicin, cisplatin, olaparib, and etoposide, while decreasing sensitivity to 5-fluorouracil.

Together, these findings identify SLC44A4 as a potential tumor-suppressive factor in NPC and suggest that SLC44A4 may serve as a biomarker for metabolic state, B-cell/TLS-associated immune features, and vulnerability to DNA damage–based therapies.”

Comment 6: There are some grammatical errors in the text Suggest the authors carefully check throughout the manuscript and revise them.

Response: We thank the reviewer for pointing this out. We have carefully checked the entire manuscript and revised grammatical errors, awkward expressions, and unclear sentences. We also improved the consistency of terminology throughout the manuscript.

Comment 7: Some references are outdated and the formats are not uniform.

Response: We thank the reviewer for this helpful suggestion. We have carefully reviewed the reference list, updated relevant citations where appropriate, and standardized the reference format according to the journal requirements.

Reviewer #2: The study aims to investigate the expression, clinical significance, and functional role of solute carrier family 44 member 4 (SLC44A4) in NPC. It specifically explores the link between this transporter and tumor suppression, DNA damage vulnerability, and the immune microenvironment. Minor recommendations for the author.

Response: We sincerely thank the reviewer for the positive evaluation of our study and for the constructive suggestions. In response to the reviewer’s minor recommendations, we have carefully revised and improved the manuscript point by point. In the revised manuscript, we have further clarified the relevant statements and added necessary explanations to enhance the accuracy and readability of the paper.

Comment 1: Validation of choline transport activity:

The authors acknowledge that while the SLC44 family is implicated in choline transport, SLC44A4's specific activity remains unproven. Experimental confirmation of its transport function would solidify the link to the "Choline metabolism in cancer" pathway.

Response: We thank the reviewer for this important suggestion. In the revised study, we performed additional qPCR analyses to examine whether SLC44A4 overexpression affects genes involved in choline/phospholipid metabolism (Figure S2). We found that PLD1 and PLCL2 were consistently upregulated in both HONE1 and SUNE1 cells following SLC44A4 overexpression, whereas other examined genes showed limited or cell line-dependent changes. These results suggest that SLC44A4 may be associated with remodeling of choline/phospholipid metabolism-related transcriptional programs in NPC cells.

However, we acknowledge that these data do not directly demonstrate choline transport activity of SLC44A4. Therefore, we have revised the manuscript to avoid overstatement and now describe SLC44A4 as being associated with choline/phospholipid metabolic remodeling rather than as a confirmed choline transporter in NPC. We have also added this point as a limitation and indicated that future studies using radiolabeled or isotope-labeled choline uptake assays, choline metabolic flux analysis, and lipidomic profiling are required to determine whether SLC44A4 directly mediates choline transport.

Comment 2: Elucidate TLS Recruitment Mechanism:

While the authors successfully demonstrate a strong correlation between SLC44A4 and TLS/B-cell infiltration, the underlying molecular mechanism remains unclear. Specifically, it is not yet defined which chemokines are secreted by SLC44A4 to drive this process. Identifying these key molecular mediators would significantly elevate the mechanistic depth of the work and better support its clinical relevance.

Response: We thank the reviewer for raising this important mechanistic question. To explore whether SLC44A4 alters the expression of chemokine genes involved in TLS formation and B-cell recruitment, we performed additional qPCR analyses in SLC44A4-overexpressing NPC cells. We examined several TLS- and immune recruitment-related chemokines, including CXCL13, CCL19, CCL21, CXCL12, CXCL9, CXCL10, and CXCL11. We found that SLC44A4 overexpression significantly increased the mRNA expression of CXCL10, which is also one of the major chemokines secreted by tumor cells. In contrast, CXCL13, CCL19, CCL21 and CXCL12, which are typical TLS/B cell-associated chemokines, did not show consistent upregulation.

These data suggest that SLC44A4 overexpression does not directly activate the classical TLS-organizing chemokine program in NPC tumor cells. Instead, SLC44A4 may be associated with a more inflammatory or immune-permissive tumor-cell state. The consistent upregulation of CXCL10 suggests a potential link with the CXCL10/CXCR3 axis, which may facilitate recruitment or functional positioning of CXCR3⁺ immune cells in certain inflammatory and tumor contexts (PMID: 37699392, PMID: 40009768, PMID: 41617729). However, CXCL10 alone is unlikely to be sufficient to organize TLS formation. Mature TLS development generally requires coordinated lymphoid-organizing modules, including CXCL13–CXCR5, CCL19/CCL21–CCR7, LTβR–NF-κB signaling, HEVs, and FDC-associated stromal programs (PMID: 39117919). CXCL10 is therefore more likely to function as an amplifier of TLS functionalization/inflammation rather than as an autonomous TLS-organizing factor. We have revised the manuscript accordingly to avoid implying a direct causal role of SLC44A4 in TLS formation and have added this point to the Discussion. The corresponding changes have been made in the revised manuscript on Page 11, Lines 273–277 and Page 14, Lines 365–376.

Attachment Submitted filename: Point-by-Point Response.docx

# Peer review: Decision Letter 1

**DOI:** 10.1371/journal.pone.0352812.r003

**Authors:** Peng Zhang, Peng Zhang

15 Jun 2026

Downregulation of SLC44A4 in nasopharyngeal carcinoma is associated with malignant progression, B-cell/TLS-related immune features, and sensitivity to DNA-damaging agents

PONE-D-26-10507R1

Dear Dr. Liu,

We’re pleased to inform you that your manuscript has been judged scientifically suitable for publication and will be formally accepted for publication once it meets all outstanding technical requirements.

Within one week, you’ll receive an e-mail detailing the required amendments. When these have been addressed, you’ll receive a formal acceptance letter and your manuscript will be scheduled for publication.

An invoice will be generated when your article is formally accepted. Please note, if your institution has a publishing partnership with PLOS and your article meets the relevant criteria, all or part of your publication costs will be covered. Please make sure your user information is up-to-date by logging into Editorial Manager at Editorial Manager® and clicking the ‘Update My Information' link at the top of the page. For questions related to billing, please contact billing support.

If your institution or institutions have a press office, please notify them about your upcoming paper to help maximize its impact. If they’ll be preparing press materials, please inform our press team as soon as possible -- no later than 48 hours after receiving the formal acceptance. Your manuscript will remain under strict press embargo until 2 pm Eastern Time on the date of publication. For more information, please contact onepress@plos.org.

Kind regards,

Peng Zhang, Ph.D.

Academic Editor

PLOS One

Additional Editor Comments (optional):

Reviewers' comments:

Reviewer's Responses to Questions

**Comments to the Author**

1. If the authors have adequately addressed your comments raised in a previous round of review and you feel that this manuscript is now acceptable for publication, you may indicate that here to bypass the “Comments to the Author” section, enter your conflict of interest statement in the “Confidential to Editor” section, and submit your "Accept" recommendation.

Reviewer #1: All comments have been addressed

**********

2. Is the manuscript technically sound, and do the data support the conclusions?

The manuscript must describe a technically sound piece of scientific research with data that supports the conclusions. Experiments must have been conducted rigorously, with appropriate controls, replication, and sample sizes. The conclusions must be drawn appropriately based on the data presented.

Reviewer #1: Yes

**********

3. Has the statistical analysis been performed appropriately and rigorously?

Reviewer #1: Yes

**********

4. Have the authors made all data underlying the findings in their manuscript fully available?

The PLOS Data policy requires authors to make all data underlying the findings described in their manuscript fully available without restriction, with rare exception (please refer to the Data Availability Statement in the manuscript PDF file). The data should be provided as part of the manuscript or its supporting information, or deposited to a public repository. For example, in addition to summary statistics, the data points behind means, medians and variance measures should be available. If there are restrictions on publicly sharing data—e.g. participant privacy or use of data from a third party—those must be specified.

Reviewer #1: Yes

**********

5. Is the manuscript presented in an intelligible fashion and written in standard English?

PLOS ONE does not copyedit accepted manuscripts, so the language in submitted articles must be clear, correct, and unambiguous. Any typographical or grammatical errors should be corrected at revision, so please note any specific errors here.

Reviewer #1: Yes

**********

6. Review Comments to the Author

Please use the space provided to explain your answers to the questions above. You may also include additional comments for the author, including concerns about dual publication, research ethics, or publication ethics. (Please upload your review as an attachment if it exceeds 20,000 characters)

Reviewer #1: All commnets that raised have completely been addressed, and the language of the manuscript has been promoted. I have not other comments.

**********

7. PLOS authors have the option to publish the peer review history of their article (what does this mean?). If published, this will include your full peer review and any attached files.

If you choose “no”, your identity will remain anonymous but your review may still be made public.

**Do you want your identity to be public for this peer review?**  For information about this choice, including consent withdrawal, please see our Privacy Policy.

Reviewer #1: No

**********

# Editor report: Acceptance letter

**DOI:** 10.1371/journal.pone.0352812.r004

**Authors:** Peng Zhang, Peng Zhang

PONE-D-26-10507R1

PLOS One

Dear Dr. Liu,

I'm pleased to inform you that your manuscript has been deemed suitable for publication in PLOS One. Congratulations! Your manuscript is now being handed over to our production team.

At this stage, our production department will prepare your paper for publication. This includes ensuring the following:

* All references, tables, and figures are properly cited

* All relevant supporting information is included in the manuscript submission,

* There are no issues that prevent the paper from being properly typeset

You will receive further instructions from the production team, including instructions on how to review your proof when it is ready. Please keep in mind that we are working through a large volume of accepted articles, so please give us a few days to review your paper and let you know the next and final steps.

Lastly, if your institution or institutions have a press office, please let them know about your upcoming paper now to help maximize its impact. If they'll be preparing press materials, please inform our press team within the next 48 hours. Your manuscript will remain under strict press embargo until 2 pm Eastern Time on the date of publication. For more information, please contact onepress@plos.org.

You will receive an invoice from PLOS for your publication fee after your manuscript has reached the completed accept phase. If you receive an email requesting payment before acceptance or for any other service, this may be a phishing scheme. Learn how to identify phishing emails and protect your accounts at https://explore.plos.org/phishing.

If we can help with anything else, please email us at customercare@plos.org.

Thank you for submitting your work to PLOS ONE and supporting open access.

Kind regards,

PLOS ONE Editorial Office Staff

on behalf of

Prof. Peng Zhang

Academic Editor

PLOS One
